# Systematic Reviews and Meta-Analyses of Home Telemonitoring Interventions for Patients With Chronic Diseases: A Critical Assessment of Their Methodological Quality

**DOI:** 10.2196/jmir.2770

**Published:** 2013-07-23

**Authors:** Spyros Kitsiou, Guy Paré, Mirou Jaana

**Affiliations:** ^1^Canada Research Chair in Information Technology in Health CareHEC MontrealMontreal, QCCanada; ^2^Telfer School of ManagementUniversity of OttawaOttawa, ONCanada; ^3^School of BusinessLebanese American UniversityBeirutLebanon

**Keywords:** meta-analysis as topic, systematic review as topic, home telemonitoring, telehealth, telemetry, quality assessment, risk of bias, chronic diseases, heart failure, diabetes, hypertension, pulmonary disease

## Abstract

**Background:**

Systematic reviews and meta-analyses of home telemonitoring interventions for patients with chronic diseases have increased over the past decade and become increasingly important to a wide range of clinicians, policy makers, and other health care stakeholders. While a few criticisms about their methodological rigor and synthesis approaches have recently appeared, no formal appraisal of their quality has been conducted yet.

**Objective:**

The primary aim of this critical review was to evaluate the methodology, quality, and reporting characteristics of prior reviews that have investigated the effects of home telemonitoring interventions in the context of chronic diseases.

**Methods:**

Ovid MEDLINE, the Database of Abstract of Reviews of Effects (DARE), and Health Technology Assessment Database (HTA) of the Cochrane Library were electronically searched to find relevant systematic reviews, published between January 1966 and December 2012. Potential reviews were screened and assessed for inclusion independently by three reviewers. Data pertaining to the methods used were extracted from each included review and examined for accuracy by two reviewers. A validated quality assessment instrument, R-AMSTAR, was used as a framework to guide the assessment process.

**Results:**

Twenty-four reviews, nine of which were meta-analyses, were identified from more than 200 citations. The bibliographic search revealed that the number of published reviews has increased substantially over the years in this area and although most reviews focus on studying the effects of home telemonitoring on patients with congestive heart failure, researcher interest has extended to other chronic diseases as well, such as diabetes, hypertension, chronic obstructive pulmonary disease, and asthma. Nevertheless, an important number of these reviews appear to lack optimal scientific rigor due to intrinsic methodological issues. Also, the overall quality of reviews does not appear to have improved over time. While several criteria were met satisfactorily by either all or nearly all reviews, such as the establishment of an a priori design with inclusion and exclusion criteria, use of electronic searches on multiple databases, and reporting of studies characteristics, there were other important areas that needed improvement. Duplicate data extraction, manual searches of highly relevant journals, inclusion of gray and non-English literature, assessment of the methodological quality of included studies and quality of evidence were key methodological procedures that were performed infrequently. Furthermore, certain methodological limitations identified in the synthesis of study results have affected the results and conclusions of some reviews.

**Conclusions:**

Despite the availability of methodological guidelines that can be utilized to guide the proper conduct of systematic reviews and meta-analyses and eliminate potential risks of bias, this knowledge has not yet been fully integrated in the area of home telemonitoring. Further efforts should be made to improve the design, conduct, reporting, and publication of systematic reviews and meta-analyses in this area.

## Introduction

The prevalence of chronic diseases such as diabetes, cardiovascular, and respiratory conditions continues to pose a significant and longstanding challenge for virtually all health care systems, requiring fundamental changes in the management and delivery of patient care [1-3]. Home telemonitoring (HT) represents a promising approach for enabling patients with chronic conditions to be followed up by clinicians more frequently, over longer periods of time, away from hospital settings [4-6]. HT is a particular form of telehealth that encompasses the use of remote access information and communication technologies (eg, telemetry devices, intelligent sensors, hand-held or wearable technologies) for the timely transmission of symptoms, physiological, and disease-related data from the patients’ home to a telemonitoring center supporting clinical decisions [4,5,7]. The underlying goal of HT is to provide doctors and nurses with accurate and timely information necessary to remotely detect any abnormal health parameters and complications associated with the disease, earlier than during a scheduled follow-up or an emergency visit. This allows timely interventions before exacerbations and complications occur, necessitating admission to the hospital and use of more resources.

Over the years, in the context of national eHealth strategies in Europe, Canada, Australia, the United States, and other parts of the world, there have been numerous efforts and research initiatives to examine the effectiveness of HT for patients with chronic diseases as a potential cost-saving approach (eg, [8,9]). The Veterans Health Administration’s extensive home telehealth service in the United States [10] and the Whole System Demonstrator (WSD) program in the United Kingdom [11] are a few examples. Nonetheless, the benefits from wider diffusion and use of HT applications have not been fully achieved yet [12]. The confidence and acceptance of health authorities to support and reimburse HT services for the management of chronic diseases depend to a large extent on the availability of reliable and robust scientific evidence from the field [13].

Systematic reviews (SRs) and meta-analyses (MAs) are powerful research tools that have been established in the health sciences, and more recently in the medical informatics field, as the cornerstone of evidence-based practice [14,15]. They adhere closely to a set of rigid scientific guidelines and use rigorous and reproducible methods to identify, select, appraise, and synthesize the results of clinical studies, in order to minimize the potential for bias in addressing a specific research question [16]. SRs and MAs have become increasingly important in the health care domain and their value to policy makers, clinicians, and researchers is well recognized [17]. When properly conducted, they provide relevant information for policy makers and serve as the foundation for the development of evidence-based practice and clinical guidelines.

However, the quality and internal validity of SRs and MAs depend on many aspects pertaining to the conduct of the review and the quality of empirical studies selected for inclusion. Flaws and deficiencies in the methods concerning the bibliographic search, selection, appraisal, and synthesis of evidence can lead to invalid conclusions with significant implications for patient care and decision makers. Hence, researchers have proposed and adopted evaluation tools that allow a close examination of the methodological rigor of reviews in several clinical areas (eg, [17-21]).

Reviews focusing on HT interventions for patients with chronic diseases have increased over the past decade. While a few criticisms about their methodological rigor and approaches have recently appeared (eg, [6,11,22,23]), no formal appraisal of their scientific quality has been conducted yet. This paper attempts to fill this gap by evaluating the methodology, quality, and reporting characteristics of SRs and MAs of HT interventions in the context of chronic diseases, in order to identify risks of bias that may have affected their internal validity. In studying and presenting methodological deficiencies identified in prior reviews, we do not intend to exemplify author incompetence. In fact, many of the authors of the included reviews are rightly acknowledged as leading experts and most of the included papers have provided the base for building evidence in a relatively recent discipline. However, we truly believe that scientific progress in this particular area of HT will not occur through the accumulation of uncontested findings, but through a continuous process of constructive criticism, vigorous debate, and creation of awareness [24]. To this end, our objective is to constructively inform other scholars and strengthen knowledge development by giving focus and direction to future reviews of HT for further improvement.

## Methods

### Inclusion and Exclusion Criteria

#### Overview

All inclusion and exclusion criteria were defined a priori. Citations identified in the search were assessed for eligibility against the study selection criteria explained below: types of studies, patients, interventions, and outcomes.

#### Types of Studies

Only prior SRs and MAs considering the effects of HT and published in peer-reviewed journals or the Cochrane Library were eligible for inclusion. To determine during the screening process whether a published article corresponded to these review types, we relied on key characteristics outlined by the Cochrane Collaboration [25]. In particular, we considered a review to be systematic if it included: (1) a set of clearly formulated research objectives or research questions with predetermined eligibility criteria for the selection of relevant empirical studies, (2) an explicit, reproducible methodology, (3) a systematic search strategy that attempted to identify all studies that would meet the eligibility criteria, and (4) a systematic presentation, analysis, and synthesis of the characteristics and findings of the included studies. Depending on the methods used to summarize and synthesize the available evidence from primary studies, systematic reviews can be classified as qualitative/narrative or quantitative (ie, meta-analyses). In our sample we included both MAs and narrative SRs. Reviews that were self-described as systematic, whether in the title, abstract, or methods of the paper, were also included. These criteria were utilized regardless of the quality or comprehensiveness of the review. We excluded conference proceedings, review summaries, editorials, and unpublished works.

#### Types of Patients and Interventions

In order to meet the inclusion criteria, the reviews had to investigate the effectiveness of HT interventions for patients with one of the following chronic conditions: congestive heart failure, hypertension, diabetes, or respiratory conditions. They also had to include primary (empirical) studies that involved the use of information and communication technologies by patients for the timely transmission and remote monitoring of vital signs (eg, arterial blood pressure, cardiac rate), biometric, and disease-related data (eg, blood glucose levels, symptoms, use of medication) from the patients’ residence to a clinician (eg, nurse, doctor, or allied health professional) at a health care service center. SRs that investigated and combined collectively (ie, without making a distinction) the effects of HT with other stand-alone multidisciplinary interventions of remote patient monitoring (eg, structured telephone support, telediagnosis, or teleconsultation) were excluded.

#### Outcomes

Prior reviews were included only if primary or secondary outcomes from the primary studies pertaining to the clinical, structural (eg, utilization of services), behavioral (eg, impacts on patients’ behavior), or economic effects of HT were synthesized and presented. Reviews that focused on other aspects such as the technical feasibility of HT modalities were excluded.

### Search Strategy

We performed a literature search on Ovid MEDLINE, the Database of Abstract of Reviews of Effects (DARE), and Health Technology Assessment Database (HTA) of the Cochrane Library (from 1966 to December 2012) in order to identify all relevant reviews. On the Cochrane Library, we conducted the search using four keywords (telemonitoring, telecare, telehealth, telehomecare). On Ovid MEDLINE, we used the same keywords in conjunction with each of the following terms: systematic review, meta-analysis, and review. Language restrictions were not applied to any of the searches.

### Selection of Relevant Reviews

**Figure 1:**
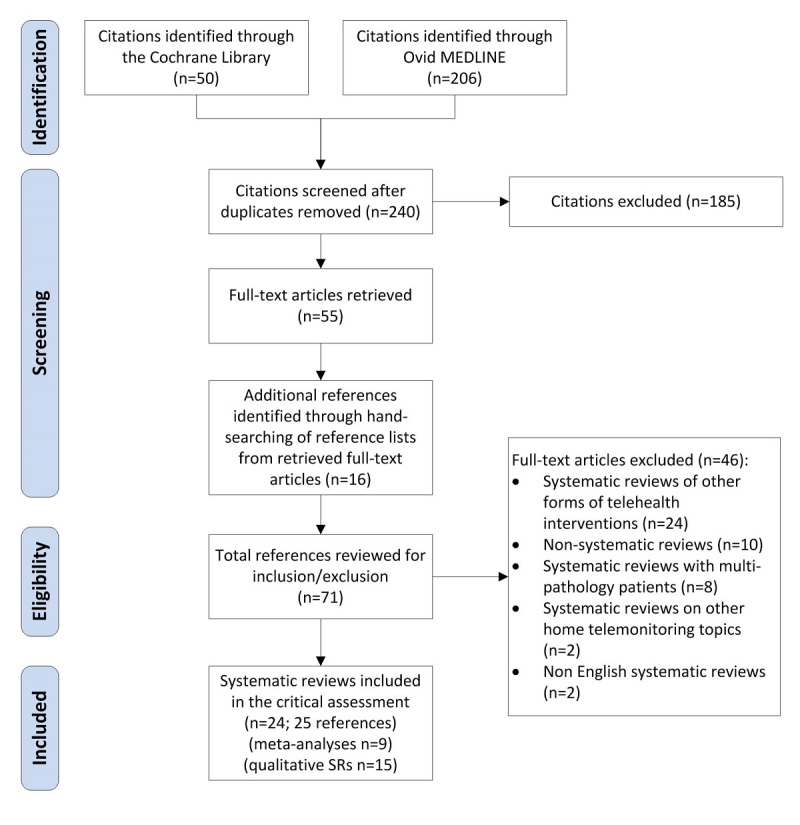
Flow diagram describing the selection process of SRs and MAs.

As shown in Figure 1, our initial search resulted in 240 references after eliminating duplicates. The title and abstract of these references were examined independently by the 3 authors to identify articles that appeared potentially relevant to this study area. Any differences were resolved by discussion until consensus was achieved. Based on the inclusion criteria, 185 references were deemed not relevant and were excluded. The remaining 55 were identified as potentially relevant, and full copies of these references were retrieved for further assessment. The reference lists of these articles were manually examined to identify potentially relevant reviews that were not originally captured in the initial search. This process yielded 16 additional references. Several reviews were excluded as they concerned other forms of telehealth interventions (n=24), they included primary studies with multipathology patients (n=8) or reviewed topics other than the effectiveness of HT (n=2). Other studies were excluded because they were not SRs or MAs (n=10), and 2 reviews were excluded as they were published in a language other than English. Multimedia Appendix 1 provides the full list of references that were excluded. The final number of SRs included in this critical review was 24 [26-49]. Note that one review was published initially as a Cochrane Collaboration review [31], and later an abridged version of it appeared in a journal [50]. In our assessment, we used the former publication as it is more detailed.

### Extraction of Information

One reviewer (SK) extracted explicit details from each review in a nonblinded manner by using an electronic extraction form that was developed for the purposes of this study. All extracted data were examined for accuracy by 2 of the reviewers (GP and MJ), and any disagreements were reconciled through consensus. The information sought included general details pertaining to the characteristics of the reviews (eg, number of authors, origin of the corresponding author, year of publication, journal characteristics, sources of funding) and more specific details about the use and interpretation of methods for synthesizing the available evidence (eg, meta-analytic and qualitative techniques).

### Assessment of Methodological Quality

The methodological quality of the 24 reviews was appraised independently in a nonblinded format by 2 reviewers (SK and GP) using the Revised Assessment of Multiple Systematic Reviews (R-AMSTAR) instrument [51]. Any disagreements were reconciled through consensus. R-AMSTAR was chosen on the basis that it is a validated instrument that offers the ability to conduct an in-depth appraisal of SRs and MAs by assessing the presence of (1) an a priori design, (2) duplicate study selection and data extraction, (3) a comprehensive literature search, (4) the inclusion of gray literature, (5) a list of included/excluded studies, (6) a profile of the included studies, (7) a documented assessment of the scientific quality of included studies, (8) the appropriate use of the scientific quality in forming conclusions, (9) the appropriate use of methods to combine findings of studies, (10) the assessment of the likelihood of publication bias, and (11) the proper documentation of conflict of interest. Each of these domains will be described in greater detail later.

## Results

### Profile of the Reviews

**Figure 2:**
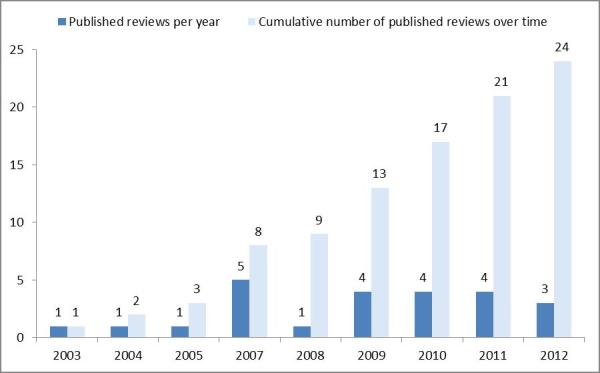
Number of HT systematic reviews and meta-analyses published per year.

Figure 2 displays the trend over time in the publication of SRs and MAs of HT interventions. Our findings reveal that the first review was published in 2003 [32]. Clearly, very few reviews were published prior to 2007. But since then, the number of HT reviews has increased substantially.

As shown in Table 1, the largest body of reviews (n=10) focused on the effects of HT on patients with congestive heart failure [26-35]. Four reviews (17%) considered patients with hypertension [36-39]; 4 reviews (17%) examined HT for patients with respiratory conditions such as chronic obstructive pulmonary disease (n=2), cystic fibrosis (n=1), and asthma (n=1) [40-43]; and 4 other reviews (17%) focused on patients with diabetes [44-47]. Last, our sample comprises 2 comprehensive SRs (8%), which investigated the effects of HT across various chronic diseases (ie, heart failure, hypertension, diabetes, and respiratory conditions) [48,49]. These reviews were included since HT effects were reported separately for each condition.

All but 3 reviews were published in peer-reviewed journals. The 3 most common sources were the *Journal of Telemedicine and Telecare* (n=3), *Telemedicine and e-Health* (n=3), and the *Journal of Evaluation in Clinical Practice* (n=2). Five reviews [27,31,42,46,48] reported being updates of previous reviews. In most articles, the corresponding authors were from North America with 10 being from Canada and 4 from the United States. Six reviews originated in Europe (4 in the United Kingdom, 1 in Greece, and 1 in Italy), 3 in Australia, and 1 in Taiwan. Six reviews comprised a multinational group of researchers.

Most reviews were conducted by 2 or more authors and only 2 [35,42] were single authored. The majority of reviews (63%) were funded by government organizations or health care agencies. Five of these received additional funding either from the industry or from academic institutions. Less than half of the reviews combined the results from the primary studies into an MA, and most reviews (63%) used qualitative approaches to synthesize the available evidence. MAs were found to be cited more frequently (mean 103.6, SD 108.2, 95% CI 13.1-194.1) than SRs (mean 61.1, SD 77.2, 95% CI 18.37-103.90), but this difference was not statistically significant (*P*=.287).

### Methodological Quality of Reviews

The results of the methodological quality of the included reviews are presented in Table 2. We outline all 41 quality criteria covered by the R-AMSTAR instrument and present the percentage of review articles that met each of them. Multimedia Appendix 2 provides a detailed analysis of each review. We list in lower-case letters all the criteria that were covered satisfactorily [51]. In the following sections, we present an analysis of the key findings within each R-AMSTAR domain.

**Table 1:** Profile of the reviews.

Chronic disease	Reference	Year	Type of Review	Number of cites^a^	Period covered	Total # of included studies (number of RCTs^d^)
**Heart failure**						
	Chaudhry et al [26]	2007	SR^b^	94	1966-2006	9 (9)
	Clark et al [27]	2007	MA^c^	323	2002-2006	5 (5)
	Clarke et al [28]	2011	MA	23	1969-2009	13 (13)
	Dang et al [29]	2009	SR	30	1966-2009	9 (9)
	Giamouzis et al [30]	2012	SR	4	2001-2011	12 (12)
	Inglis et al [31]	2010	MA	173	2006-2008	14 (14)
	Louis et al [32]	2003	SR	199	1966-2002	24 (6)
	Maric et al [33]	2009	SR	53	up to 2007	41 (12)
	Polisena et al [34]	2010	MA	50	1998-2008	21 (11)
	Seto [35]	2008	SR	48	up to 2007	8 (4)
**Hypertension**						
	AbuDagga et al [36]	2010	SR	18	1995-2009	15 (10)
	Jaana et al [37]	2007	SR	13	1966-2006	14 (3)
	Omboni et al [38]	2011	MA	7	up to 2010	12 (12)
	Verberk et al [39]	2011	MA	6	not reported	9 (9)
**Respiratory conditions**						
	Bolton et al [40]	2011	SR	16	1990-2009	6 (2)
	Cox et al [41]	2012	SR	1	1998-2011	8 (1)
	Franek et al [42]	2012	SR	4	2000-2010	5 (3)
	Jaana et al [43]	2009	SR	49	1966-2007	14 (3)
**Diabetes**						
	Farmer et al [44]	2005	MA	127	1966-2004	26 (16)
	Jaana et al [45]	2007	SR	70	not reported	17 (11)
	MAS [46]	2009	MA	-	2007-2009	8 (8)
	Montori et al [47]	2004	MA	120	1982-2003	8 (8)
**SRs covering various chronic diseases**						
	Paré et al [48]	2010	SR	44	1966-2008	CHF: 17 (13); Hypertension: 13 (5); Asthma: 8 (6); Diabetes: 24 (21)
	Paré et al [49]	2007	SR	274	1990-2006	CHF: 16 (7); Hypertension: 14 (3); Respiratory Conditions: 18 (4); Diabetes: 17 (12)

^a^According to *Google Scholar* as of March 28, 2013.

^b^SR: Narrative/Qualitative systematic review.

^c^MA: Meta-analysis.

^d^randomized controlled trials.

**Table 2:** Percentage of reviews that satisfactorily met each R-AMSTAR criterion.

Criterion	Description	Yes, %
Q 1.a	The design of the study was established before the conduct of the review (ie, a priori design).	100
Q 1.b	There was a statement of inclusion criteria.	100
Q 1.c	There was a PICO research question/statement.	67
Q 2.a	There were at least 2 independent data extractors as stated or implied.	42
Q 2.b	There was a statement of recognition or awareness of consensus procedure for disagreements.	46
Q 2.c	Disagreements among extractors were resolved properly as stated or implied.	38
Q 3.a	At least 2 electronic sources were searched (eg, Medline and EMBASE).	96
Q 3.b	The report includes years and databases searched.	92
Q 3.c	Key words and/or MESH terms are stated.	92
Q 3.d	In addition to the electronic databases (PubMed, EMBASE, Medline), the search was supplemented by consulting current contents such as reviews, textbooks, specialized registers, or experts in the particular field of study or by reviewing the references in the studies found.	79
Q 3.e	Journals were “hand searched” or “manual searched” (ie, identifying highly relevant journals and conducting a manual, page-by-page search of their entire contents looking for potentially eligible studies).	13
Q 4.a	The authors stated that they searched for reports regardless of publication type.	8
Q 4.b	The authors state whether or not they excluded any reports (from the systematic review), based on their publication status, language, etc.	83
Q 4.c	“NonEnglish” papers were translated.	4
Q 4.d	There was no language restriction or recognition of nonEnglish articles.	21
Q 5.a	Table/list/or figure of included studies was provided; a reference list does not suffice.	92
Q 5.b	Table/list/or figure of excluded studies was provided either in the article or in a supplemental source (ie, online). (Excluded studies refers to those studies seriously considered on the basis of title and/or abstract, but rejected after reading the body of the text.)	25
Q 5.c	Author satisfactorily/sufficiently stated the reason for exclusion of the seriously considered studies.	63
Q 5.d	Reader is able to retrace the included and the excluded studies anywhere in the article bibliography, reference, or supplemental source.	25
Q 6.a	The characteristics of the included studies are provided in an aggregated form such as a table, data from the original studies were provided on the participants, interventions AND outcomes.	88
Q 6.b	The authors provided the ranges of relevant characteristics in the studies analyzed (eg, age, race, sex, relevant socioeconomic data, disease status, duration, severity, or other diseases are reported).	83
Q 6.c	The information provided appears to be complete and accurate (ie, there is a tolerable range of subjectivity here. Is the reader left wondering? If so, state the needed information and the reasoning).	88
Q 7.a	A priori methods of assessment were provided (eg, for effectiveness studies if the author(s) chose to include only randomized, double-blind, placebo controlled studies, or allocation concealment as inclusion criteria); for other types of studies alternative items will be relevant.	38
Q 7.b	The scientific quality of the included studies appears to be meaningful (ie, a scale such as High, Low or A, B, C is used).	33
Q 7.c	Discussion/recognition/awareness of level of evidence	21
Q 7.d	Quality of evidence was rated/ranked based on characterized instruments (Characterized instrument is a created instrument that ranks the level of evidence, eg, GRADE).	21
Q 8.a	The results of the methodological rigor and scientific quality were considered in the analysis and the conclusions of the SR.	25
Q 8.b	The results of the methodological rigor and scientific quality were explicitly stated in formulating recommendations.	25
Q 8.c	To have conclusions integrated/drives towards a clinical consensus statement.	n/a
Q 8.d	This clinical consensus statement drives toward revision or confirmation of clinical practice guidelines.	n/a
Q 9.a	The authors provided a statement of criteria that were used to decide that the studies analyzed were similar enough to be pooled.	0
Q 9.b	For the pooled results, a test was performed to ensure the studies were combinable, to assess their homogeneity (ie, Chi-square test for homogeneity, I^2^).	38
Q 9.c	There was a recognition of heterogeneity or lack of thereof.	38
Q 9.d	If heterogeneity existed a “random effects model” was used and/or the rationale (ie, clinical appropriateness) of combining was taken into consideration (ie, was it sensible to combine), or stated explicitly.	25
Q 9.e	If homogeneity existed, the authors stated a rationale or a statistical test.	0
Q 10.a	Recognition of publication bias or file-drawer effect.	21
Q 10.b	Assessment of publication bias included graphical aids (eg, funnel plot, other available tests).	13
Q 10.c	Statistical tests (eg, Egger regression test).	0
Q 11.a	The authors provided a statement of sources of support.	79
Q 11.b	There was no conflict of interest.	50
Q 11.c	The authors provided an awareness/statement of support or conflict of interest in the primary inclusion studies.	4

### A Priori Design (Q1)

All reviews included in our sample established their review design (Q1.a) and the criteria of eligibility for the selection of studies (Q1.b) before commencing with the search, collection, and data abstraction. However, most reviews suffered from a lack of clarity in framing their research questions/objectives according to the “PICO” framework (Population, Intervention, Comparison, Outcomes) recommended by methodologists and the PRISMA statement [16,52]. Although the patient population or chronic disease and the intervention under scrutiny were stated explicitly in all of the included reviews, the comparator (control) group and the outcomes of the intervention being assessed were specified in fewer cases: 25% and 67% respectively. Well-formulated research objectives addressing all 4 PICO components were identified in just 3 review articles (15%). Overall, a majority (67%) of reviews reported the patient population, the intervention, and the clinical outcomes of interest and, hence, was judged as having covered item Q1.c satisfactorily.

### Duplicate Study Selection and Data Extraction (Q2)

The screening process for the selection of primary studies was performed in most cases (67%) independently, at least by 2 reviewers. Nevertheless, data extraction from the primary studies was reported as being performed independently and in duplicate in less than half of the reviews (Q2.a). In assessing the accuracy of data abstraction against primary studies in at least a sample of the included reviews as suggested by methodologists [53], we detected an instance of inappropriate coding in 1 MA [28] between the extracted data and the original publication of 1 randomized controlled trial (RCT) [54] for the outcome of congestive heart failure hospital admission. The total number of events between the control and experimental group was recorded reversely. As such, the estimated summary effect appears slightly higher and the I^2^ point estimate for heterogeneity deflated (RR 0.73 [0.62-0.87] *P*=.0004; I^2^=0 vs RR 0.78 [0.65-0.93] *P*=.004; I^2^=46%). Data extraction was not reported being duplicated in this MA.

Out of the 24 reviews, 11 (46%) stated whether there was a consensus procedure in place or a third reviewer to resolve any disagreements (Q2.b), and 9 (38%) included a statement regarding proper resolution of existing disagreements among the reviewers (Q2.c). Overall, as shown in Multimedia Appendix 2, only one third of the reviews covered satisfactorily all of the criteria included in this domain. Additional information pertaining to the methods employed during data extraction, such as use of piloted forms/coding sheets, steps undertaken to avoid double counting of duplicate published reports, and methods used to collect additional information from the authors of the original studies were scarce.

### Search Comprehensiveness (Q3)

Analysis of domain 3, which consisted of 5 criteria, showed that almost all reviews (96%) used at least 2 electronic databases to search for primary studies (Q3.a). The most prevalent databases were Medline (100%), the Cochrane Library (70%), and EMBASE (60%). All in all, 22 reviews (92%) reported the years and databases searched (Q3.b); 22 (92%) stated the keywords that were used (Q3.c); and 19 (79%) stated that the search was supplemented by reviewing the references in the studies found (Q3.d). A manual search of highly relevant journals to identify eligible studies was performed in only 3 (13%) reviews (Q3.e). Fourteen reviews (58%) used a QUOROM/PRISMA flow chart to depict and describe graphically the sequence of steps undertaken for the search and selection of relevant articles. However, presentation of the full electronic search strategy for at least 1 major database—so that one could repeat the search or assess its comprehensiveness—was made available in only 5 reviews (21%). As shown in Multimedia Appendix 2, only 2 reviews (8%) covered satisfactory all 5 criteria of the R-AMSTAR instrument within this particular domain.

### Inclusion of Gray Literature (Q4)

Interestingly, most reviews focused on peer-reviewed primary studies published in English language journals. Out of the 24 articles in our database, only 2 (8%) considered the inclusion of gray literature and searched for primary studies regardless of their publication type (Q4.a). In 20 reviews (83%), the authors stated that they excluded primary studies based on their publication status (eg, abstracts, conference proceedings, and language) (Q4.b). Only one review (4%) reported that nonEnglish papers were translated (Q4.c), while 5 (21%) reported that no language restrictions were applied to the search and inclusion of studies (Q4.d).

### Included and Excluded Studies Provided (Q5)

Most reviews (92%) presented a list of included studies (Q5.a), but only 25% reported a list of excluded studies in the article or in a supplement source (eg, online appendix) (Q5.b). Hence, retracing both the included and excluded studies was feasible in only 6 reviews (Q5.d). In 15 articles (63%), the authors explicitly reported the primary reasons for excluding studies (Q5.c) and subsequently reported the number of articles that were associated with each exclusion criterion. The latter item was covered satisfactory mainly by reviews that provided a PRISMA-like flow diagram [52].

### Characteristics of the Included Studies (Q6)

Study-level data from the original empirical studies on the participants, interventions, and outcomes were presented in an aggregated form such as a table in 21 reviews (88%) (Q6.a). Tabulated information appeared to be complete in all of them (Q6.c). In 20 reviews (83%), the authors included in the table the ranges of the relevant PICO characteristics from the primary studies (eg, mean age of patients, duration of follow-up, severity of disease) (Q6.b).

### Quality Assessment of the Primary Studies (Q7)

The methodological quality or risk of bias of the primary studies was formally appraised in 9 out of the 24 reviews (38%). In all of these, the authors provided a priori methods of assessment either in the form of a quality scale/checklist with composite scores or in the form of predefined risk of bias criteria (Q7.a). All in all, 8 reviews (33%) documented the final results of the quality appraisal in a meaningful format for each study, that is, in the form of a grade/score or total number of criteria covered satisfactorily by each review (Q7.b). In one particular review [40], the authors stated that a risk of bias assessment was conducted according to the Cochrane Collaboration criteria, yet the results of the appraisal for each individual study were not documented. Out of the 9 reviews that assessed the quality of the primary studies, only 5 rated the level of evidence across studies or outcomes according to study design (eg, RCT, observational) and scientific quality or risk of bias of the individual studies (Q7.c). All 5 reviews (21%) used various characterized instruments to rate the overall quality of evidence (Q7.d). The most prevalent was the GRADE instrument, which was used in 3 reviews.

Tables 3 and 4 summarize the different methods, instruments, and strategies ([55-61]) used in each review to assess the quality of the included primary studies and the overall quality of the evidence. Based on the combination of these approaches, we classified the reviews under two main clusters. The first cluster focused on assessing the methodological quality of each study but did not consider the overall quality of the evidence, while the second cluster performed both assessments. Quality of evidence takes into consideration the internal validity assessment (quality or risk of bias) and design of the included studies (eg, RCT, observational), as well as other potential aspects (eg, consistency and directness of results) to rate or indicate the extent to which we can be confident that the estimated effect size or the final conclusions of the review about the effectiveness of the HT intervention are correct across each outcome of interest or individual study [55].

**Table 3:** Methods and instruments used for the quality assessment of the primary studies—Cluster 1.

Cluster 1	Chaudhry 2007 [26]	Clark 2007 [27]	Cox 2012 [41]	Farmer 2005 [44]
Focus of the assessment	Study design (D) Study quality (Q)	Study design (D) Study quality (Q)	Study quality (Q)	Study quality (Q)
(Focus of the assessment) Methods of assessment	(D) Inclusion of RCTs only (Q) Jüni scale [56] and York Centre criteria [57]	(D) Inclusion of RCTs only (Q) Cochrane criteria [58]	(Q) Downs and Black scale [59]	(Q) Jadad scale [60]; used only for the assessment of RCTs
Number of Assessors	NR^a^	2	2	NR
Assessors Blinded?	NR	NR	NR	NR
Adjudication or consensus procedure	NR	Yes	Yes	NR
Cross-tabulation of results for each study by domain	No	Yes	Yes	No
Overall study quality score	Yes	N/A^b^	Yes	Yes

^a^NR: not reported.

^b^N/A: non-applicable.

**Table 4:** Methods and instruments used for the quality assessment of the primary studies—Cluster 2.

Cluster 2	Bolton 2011 [40]	Franek 2012 [42]	Inglis 2010 [31]	Polisena 2010 [34]	MAS 2009 [46]
Focus of the Assessment	Study quality (Q) Quality of evidence (E)	Study quality (Q) Quality of evidence (E)	Study design (D) Study quality (Q) Quality of evidence (E)	Study quality (Q) Quality of evidence (E)	Study design (D) Study quality (Q) Quality of evidence (E)
(Focus of the Assessment) Methods of assessment	(Q) Cochrane criteria [58] (E) Oxford Centre for Evidence-based Medicine – Levels of Evidence	(Q) Adaptation of CONSORT statement checklist for RCTs (E) GRADE [55]	(D) Inclusion of RCTs only (Q) Cochrane criteria [58] (E) GRADE [55]	(Q) and (E) Adaptation of Hailey et al instrument [61]	(D) Inclusion of RCTs only (Q) Adaptation of the levels of evidence hierarchy proposed by Goodman (E) GRADE [55]
Number of Assessors	2	NR	2	2	NR
Assessors Blinded?	NR^a^	NR	NR	NR	NR
Adjudication or consensus procedure in place	Yes	NR	NR	NR	NR
Cross-tabulation of results for each study by domain	No	Yes	Yes	No	Yes
Overall study quality score	N/A^b^	N/A	N/A	Yes	N/A
Quality of evidence ranking	Across studies	Across outcomes	Across outcomes	Across studies	Across outcomes

^a^NR: not reported.

^b^N/A: non-applicable.

It should be noted that besides the reviews that formally appraised the quality or risk of bias of the primary studies by means of an instrument, 3 additional reviews [29,43,48] used a rating scale [62] to judge the strength of evidence of the included studies. According to this scale, the strength of evidence can be determined and appropriately ranked in 1 of 9 hierarchical levels—appearing in descending order—after considering 2 important elements: (1) the type of the design employed in each primary study (eg, large RCT, small RCT, cohort), and (2) the validity of the study based on a set of conditions of scientific rigor, including study quality. However, none of the 3 reviews conducted or considered the latter component recommended by the aforementioned scale. In the context of the analysis and formulation of conclusions, all 3 reviews ranked the evidence hierarchically according to the study design “label” of each study only. They did not critically appraise or take into consideration the actual *features* of the individual studies, which ultimately influence the risk of bias. Hence, large and small-sample RCTs were ranked higher on the hierarchy of evidence compared to nonrandomized controlled trials, cohort studies, and so on.

### Scientific Quality of Included Studies Used Appropriately in Formulating Conclusions (Q8)

Out of the 9 reviews that formally assessed the scientific rigor of the primary studies (see Q7), 6 factored the results of the methodological quality into the final conclusions (Q8.a) and recommendations made for future research studies (Q8.b). Altogether, 75% of the reviews reached conclusions about the effectiveness of HT for chronic patients without considering or reflecting the potential risks of bias in the included studies. Importantly, none of the included reviews incorporated the results of the quality assessment (items in Q7) into the actual analyses of the review to explore how conclusions might be affected if studies at high risk of bias were included or excluded from the analysis.

### Appropriateness of Methods Used to Combine Studies’ Findings (Q9)

A majority of reviews in our database (63%) aggregated the results from the primary studies qualitatively, using narrative synthesis. However, the rationale behind the selected approach and the methods that the authors used to guide their decision were not generally mentioned. Out of 15 narrative SRs, 8 (53%) provided a statement as to why a qualitative synthesis of the evidence was chosen over a meta-analysis [26,29,33,37,40-42,48]. The primary reason in all of these reviews revolved generally around the existence of “heterogeneity” between the included studies. Nevertheless, the methods, criteria, or specific rules (eg, logic models based on the PICO framework) that were used to objectively support that a meta-analysis was not appropriate or sensible because the primary studies were clinically or methodologically too diverse, were not specified.

Out of the 15 SRs, only 3 (20%) provided an analysis plan with information about the methods, tools, or general framework that was used at each stage of the synthesis process [26,29,48]. In the remaining reviews, the logic of the decision-making process and the criteria based on which the authors assigned weights to the primary studies to arrive at final conclusions, were not specified. Moreover, the vast majority (93%) of SRs summarized and synthesized the available evidence using variants of raw data as reported in the original studies (eg, percentages, mean differences, *P* values, and counts). Only one [26] transformed the extracted data into a common statistical measure (eg, risk ratios) to allow for more transparent and direct comparisons between the observed treatment effects of the primary outcomes of interest.

As shown in Table 5, the authors of SRs used four distinct approaches to organize and synthesize the available evidence qualitatively. The most commonly used approach (in 10 SRs) was the “reported outcomes” method, in which analysis and synthesis of the results was carried out based on the most frequent outcomes assessed and reported in the original studies. Four reviews used a “levels of evidence” approach, in which the study design of the included studies was used as a basis to stratify and present the available evidence in descending order (eg, large RCTs, small RCTs, cohort studies, and case-control studies). Two of these coupled the “levels of evidence” with the “reported outcomes” method, while a third one used “vote counting” to present the direction of the intervention effect in each study (eg, positive, negative, and conflicting evidence for effect). In two of the SRs that we examined, the authors grouped and analyzed studies according to the primary mode of the telemonitoring intervention (eg, automated monitoring of signs and symptoms and telephone touch-pad-based HT modalities).

Out of the 24 reviews, 9 combined the findings from the primary studies quantitatively using meta-analytic methods. However, none of the MAs stated explicitly what criteria were used in the context of the research question(s) being addressed to support objectively that the HT trials analyzed were clinically and methodologically similar enough to be combined quantitatively (Q9.a). In one MA [34], it was stated that the quantitative pooling of study results was deemed inappropriate whenever substantial statistical heterogeneity (I^2^ ≥50%) was found and this heterogeneity could not be explained by means of subgroup analysis. However, from a methodological point of view (as described later), excessive reliance on I^2^ can be particularly misleading and hence, using statistical heterogeneity and point estimates of I^2^ alone as the only criterion for deciding whether an MA is appropriate or not is a rather problematic strategy [63-65]. The decision to pool and present treatment estimates in an MA is not amenable to statistical tests and should be based on the clinical and methodological relevance of any heterogeneity present (eg, the age of patients, severity of disease, duration of follow-up, technology used, and study design).

As shown in Table 5, the summary statistics of the effect measures that were used in each MA were generally related to the type of investigated outcomes and available data in the original trials (ie, dichotomous, count, or continuous). The consistency of HT effects across studies was assessed and quantified for each outcome of interest in all MAs by means of a formal statistical test (Q9.b). The most common method found in 8 MAs (Table 5) involved use of the I^2^ statistic, which is derived from the Chi-square test (Cochran’s Q statistic). With the exemption of one [39] that reported only the range of the calculated I^2^ estimates, the remaining MAs reported the precise results within the forest plots or the text of the article and provided an interpretation of the heterogeneity estimate for each investigated outcome (Q9.c).

The I^2^ statistic [66,67] measures the approximate proportion of total variability in a set of treatment effect estimates that is attributable to real clinical or methodological differences between the included studies, rather than sampling error. It takes values from 0 to 100% and often thresholds (eg, 25%, 50%, and 75%) are used to make inferences about the magnitude of inconsistencies between the findings of trials [67,68]. However, simulations have shown that the I^2^ statistic suffers from similar power and precision shortcomings as the Q statistic [64,65]. Thus, it can yield unreliable estimates in MAs that include a small number of trials (eg, k<15) with poor precision (ie, small number of patients and events). To this end, relevant guidelines [68] and methodologists [64,66,67,69,70] suggest that researchers should investigate, present, and consider in the interpretation of the results the 95% confidence interval (CI) of the I^2^ estimate, in order to adequately reflect the uncertainty (strength of evidence) around it. That is, the spectrum of possible degrees of genuine differences between the trials in terms of treatment effects. However, none of the MAs in our database reported carrying out this statistical procedure. Although the number of included HT trials was consistently lower than 12 and most trials exhibited poor precision due to the small number of registered patients, inferences about the consistency or inconsistency of HT effects across the included trials were based on I^2^ point estimates alone.

Given the potential negative implications of this methodological limitation for the reliability of MAs with respect to the interpretation of the results and choice of statistical model [70,71]), we sought to conduct a post hoc analysis to evaluate empirically the extent of uncertainty in the provided heterogeneity (I^2^) estimates. As recommended [69], we used for all calculations the noncentral χ^2^ based approach, which is implemented in the heterogi module of Stata (version 12.1) [72]. In total, we were able to calculate the I^2^ statistic and its associated 95% CIs for all but one MA [39], for a total of 22 outcomes with 4 or more studies. Based on careful appraisal of the application and interpretation of the statistical methods used in each MA, we identified the following methodological issues.

In 6 MAs [27,28,31,34,44,47] in which the I^2^ statistic was estimated to be equal to 0% for a specific outcome (Table 6), a common inference was that no heterogeneity exists or that heterogeneity is low between trials. As such, the direction and dispersion of the magnitude of clinical HT effects were interpreted as being consistent across the included trials. However, the 95% CIs, which reflect the uncertainty around these heterogeneity estimates, are particularly wide in all of these MAs, ranging from low to high heterogeneity. As shown in Table 6, the upper limits of the 95% CI crossed into the range of large heterogeneity (I^2^≥50%) in all of them and in 3 MAs it also exceeded or reached the 75% range (substantial heterogeneity), while the low limits of the intervals were always as low as 0%. This indicates that any strong inferences and conclusive statements about the similarity or comparability of the studies’ results would be difficult to make with certainty due to the general lack of evidence. Given the poor precision of the trials included in all of these MAs, it is possible that the I^2^ estimate was masked and deflated [73]. Hence, the presence of some moderate or even considerable heterogeneity between HT trials should not have been ruled out or underestimated.

The second methodological issue we identified was associated with the opposite problem, that is, overestimation of heterogeneity. In 5 forest plots of 4 MAs [28,38,46,47], in which the point estimate of I^2^ was moderate (eg, 33.8%) or quite large (eg, I^2^≥50%) (Table 6), a common inference was that there is high or even substantial inconsistency across the HT effect sizes of the trials due to genuine differences. However, as shown in Table 6, in all of these MAs the low limit of the 95% CI in the I^2^ point estimates crosses into the range of little heterogeneity (I^2^≤25%), reflecting that the evidence for large heterogeneity may not be strong enough to support the importance of the observed I^2^ value. Overestimation of heterogeneity and undue reliance on I^2^ estimates prompted researchers in one MA [47] to exhaust all possibilities of subgroup analysis and succumb to a poorly supported post hoc analysis in a quest for the causes of heterogeneity, while in another review it prevented the authors from carrying out an MA [34].

Last, a slightly more subtle, but yet important, methodological error concerns the issue of overweighting a study in an MA by double counting its study groups [24,74,75]. Specifically, one MA in our database [38] that compared the effects of HT with usual care on patients with hypertension, included in its sample an RCT [76] that had 1 control group (usual care with 247 patients) and 2 intervention groups: (1) blood pressure HT with Web training services (246 patients), and (2) blood pressure HT with pharmacist-assisted care via Web communications (237 patients). The way that the authors chose to handle this particular trial in their MA, for all reported outcomes, was to include it twice in each forest plot by double counting its control arm. However, the effect of this was that this particular trial was overpowered. It was counted once with 493 patients and once with 484 patients. As a result, its effective sample size appears to be 977 when in fact the true sample size was 730. This poses an important validity threat in the results of this particular review, as this trial was assigned considerable weight in all forest plots for the outcomes of interest.

With respect to the statistical model used, 6 MAs (67%) carried out random effects analyses, while 3 carried out fixed effect analyses (Q.9c). Two of latter studies [28,31] used the fixed-effect model even though some evidence of potentially moderate (eg, I^2^>30%) to substantial (eg, I^2^>75%) heterogeneity between studies was present. However, it was not justified why the fixed effect model was still deemed appropriate. In most reviews the rationale, criteria, or general assumptions that guided researchers in selecting one of two statistical models were not specified. Out of the 9 MAs, only 2 (22%) provided an explicit statement to justify the statistical model that was used to calculate the summary effects [27,31]. Both reviews were authored by the same group of researchers and focused on the effects of HT and structured telephone support (separately) versus usual care on patients with congestive heart failure. Interestingly, however, the selected model was different in each review, although the reasons or assumptions stated by the authors were almost identical.

### Publication Bias (Q10)

The three criteria included in this question focus on the meta-analytic methods used to assess the likelihood of publication bias, that is, the publication or nonpublication of research findings depending on the direction of the results of the primary studies. Out of the 9 MAs included in our review, 5 considered publication bias in their assessments (Q10.a) and only 3 presented the actual funnel plots in the published article (Q10.b). In these 5 MAs, authors relied on visual inspection and interpretation of funnel plots. Formal statistical tests to assess presence of bias (eg, Egger regression test) were not used by any of the MAs (Q10.c). This is reasonable, given the small number of trials included in each review. Such tests theoretically require a considerable number of primary studies for sufficient power to detect bias; a criterion that is rarely fulfilled. However, none of the MAs acknowledged the great risk of subjectivity that is associated with visual inspection of funnel plots [70,77] and the inadequacy of this method to detect bias (let alone publication bias) when the number of studies is small (eg, k<10) or when heterogeneity is significant [78,79]. As a result, in all cases, statements about the existence of strong publication bias or absence thereof were stronger than the evidence allowed.

### Conflicts of Interest (Q11)

Most reviews in our sample (79%) disclosed explicitly all the sources of support received for the conduct of the review. In 50% of them, at least one or more of the investigators were either directly affiliated or had other active involvement with entities that have competing interests in the results of the respective review, such as HT solution providers (Q10.b). Only one review (4%) examined and reported whether authors of the included empirical studies had a potential conflict of interest (Q10.c).

**Table 5:** Methods used in SRs and MAs to synthesize the available evidence from the primary studies.

Methods		Reviews	n
**Qualitative methods** (n=15)			
	Reported outcomes	[30,32,35-37,40-43,45,49]	11
	Levels of evidence (study design)	[29,32,37,48]	4^a^
	Vote counting (intervention effect)	[29]	1^a^
	Telemonitoring modality	[26,33]	2
**Meta-analytic methods** (n=9)			
**Summary statistics**			
	Risk ratios (for dichotomous data)	[27,28,31,34]	4
	Risk difference (for dichotomous data)	[27]	1^b^
	Mean difference (for continuous data)	[38,39,46]	3
	Standardized mean difference (for continuous data)	[44,47]	2
**Heterogeneity**			
	Assessment of heterogeneity by means of a statistical test	[27,28,31,34,38,39,44,46,47]	9
	Reported Cochran’s Q statistic (Chi-square test) of heterogeneity	[27,28,31,44,46,47]	6
	Reported I^2^ test of heterogeneity	[27,28,31,34,38,39,46,47]	8
**Statistical model**			
	Random effects meta-analysis	[27,34,38,47]	4
	Fixed effect meta-analysis	[28,31,44]	3
**Meta-analysis diagnostics**			
	Subgroup analysis	[34,46,47]	3
	Sensitivity analysis	[31,38]	2

^a^Includes reviews that used two different methods.

^b^Same review that used two different summary statistics.

**Table 6:** Confidence intervals for the I^2^ estimates of MAs.

	Author (Year)	Number of trials	I^2^	Low interval (95% CI)	High interval (95% CI)	Statistical model	Assessed outcomes
**Heart failure**							
	**Clark 2007 [27]**	5	0	0	79	Random effects	All-cause mortality
	**Clarke 2011 [28]**						
		10	51	0	76	Fixed effect	All-cause mortality
		6	59	0	83	Fixed effect	All-cause hospitalization
		6	0	52	75	Fixed effect	CHF-related hospitalization
		4	82	0	93	Fixed effect	All-cause emergency visits
	**Inglis 2010 [31]**						
		11	0	0	60	Fixed effect	All-cause mortality
		8	0	56	68	Fixed effect	All-cause mortality follow-up period >6 months
		8	78	70	89	Fixed effect	All-cause hospitalization
		6	85	0	93	Fixed effect	All-cause hospitalization follow-up period >6 months
		4	39	0	79	Fixed effect	CHF-related hospitalization
		4	39	0	79	Fixed effect	CHF-related hospitalization follow-up period >6 months
	**Polisena 2010 [34]**						
		6	0	0	75	Random effects	All-cause mortality
		4	5	0	85	Random effects	All-cause hospitalization
**Hypertension**							
	**Omboni 2011 [38]**						
		11	65.8	15	82	Random effects	Systolic blood pressure changes
		11	56.6	44	78	Random effects	Diastolic blood pressure changes
		6	77.9	50	91	Random effects	Blood pressure control
		5	79.1	50	91	Random effects	Number of antihypertensive drugs
**Diabetes**							
	**Farmer 2005 [44]**	9	0	20	65	Fixed effect	Glycemic control - Changes in HbA1c
	**MAS 2009 [46]**						
		7	65	0	84	Random effects	Glycemic control - Changes in HbA1c (All studies)
		4	45	0	82	Random effects	Glycemic control - Changes in HbA1c (subgroup analysis)
	**Montori 2004 [47]**						
		8	33.8	0	71	Random effects	Glycemic control - Changes in HbA1c
		7	0			Random effects	Glycemic control - Changes in HbA1c (post-hoc subgroup analysis)

## Discussion

### Principal Findings

This critical review presents the first formal and comprehensive quality assessment of published reviews that have studied the effects of HT on patients with chronic conditions. We applied the R-AMSTAR instrument to critically examine the methodological rigor and reporting characteristics of each review and also conducted a careful evaluation within the 11 domains of this particular instrument to identify risks of bias (ie, systematic errors) in inferences or results that may have affected their internal validity. To this end, R-AMSTAR was used as a general framework that guided and supported our assessment rather than a specific tool for calculating quality scores for each review. Such scores may not always reflect the true scientific quality of each review and evidence suggests that their use can be problematic in judging whether or not to trust an individual analysis, due to the potential existence of false positives or negatives [58].

The results of our bibliographic search indicate that SRs and MAs in this domain are fairly new compared to other clinical areas (eg, [18,80]). The first review was published in 2003 and focused on patients with congestive heart failure. Since then, and particularly over the last 6 years, the number of published reviews has increased substantially, while also the focus of reviewers has extended to include other chronic diseases such as chronic obstructive pulmonary disease, hypertension, and diabetes. Nonetheless, the largest body of reviews continues to focus on patients with congestive heart failure.

Based on our assessment, we found that with the recent increase in reviews of HT interventions an important number of these articles appear to lack optimal scientific rigor due to intrinsic methodological issues. Furthermore, their overall quality does not appear to have improved over time. Despite the wide availability and dissemination of important methodological guidelines [52,81] that can be utilized to guide the systematic review process and eliminate potential risks of bias, it appears that this knowledge has not yet been fully integrated in the field of HT. While several criteria were met satisfactorily by all or most reviews (eg, establishment of an a priori design (100%), reporting of inclusion/exclusion criteria (100%) and characteristics of studies (88%), use of multiple electronic searches and databases (96%)), there were other important areas that needed improvement. These areas should be considered by future SRs and MAs, in order to advance scientific progress and improve the rigor of research in the rapidly growing field of HT. As indicated by the application of the R-AMSTAR instrument and our analysis, many reviews did not perform key methodological procedures to reduce the risk of bias (eg, duplicate data extraction (42%), inclusion of gray (8%) and nonEnglish literature (21%), methodological quality assessment of included studies (38%)), and some reviews suffered from limitations in the synthesis of study results that may have affected the validity of their results and conclusions. We explain below the potential implications of these issues and provide recommendations for future reviews in this area.

### Search Strategy

Although the majority of reviews used more than 2 electronic databases to search for relevant studies, other important approaches to minimize bias and enhance the search strategy were rarely used. Only 2 reviews attempted to identify primary studies in the gray literature and the vast majority restricted all searches to English articles only, although it has been demonstrated that bias can be introduced in SRs and MAs focusing exclusively on English language publications [82,83]. Inclusion and exclusion criteria established a priori for the selection of primary studies were reported explicitly in all reviews, but most failed to provide a list of references with the studies that were excluded, as recommended by methodologists and the PRISMA statement [16,52]. These methodological issues suggest a potentially limited review of the available evidence and high risk of selection and language bias. A bibliographic analysis of citation patterns that we performed confirms these concerns. Indeed, the vast majority of reviews included in our database fell short in their identification of published studies due to various languages, publication type, and date restrictions applied in the search process. The Cochrane review by Inglis et al [31], which performed the most comprehensive search among the other SRs and MAs on heart failure, provides concrete evidence of this (Multimedia Appendix 3). Concretely, these authors identified 3 relevant trials, 2 of which were published in a language other than English (one in German and one in Italian). The German publication, which was peer-reviewed, was the largest RCT (502 patients) among all trials identified by the other reviews. Nevertheless, it was not included in any of the other reviews published after 2007, neither were the other 2 RCTs that were published as abstracts, because almost all reviews restricted their search to English publications and did not consider gray literature. To minimize the risk of selection and language bias, future reviews of HT should avoid applying such restrictions as these do not align with the notion of SRs and MAs, which aim to provide a thorough and unbiased overview of all the available empirical evidence.

### Discrepancies in the Inclusion of HT Studies

HT as a research area has witnessed considerable growth over the past decade. Nevertheless, from a conceptual point of view there seems to be a lack of consensus between authors of SRs and MAs in the terminology they used (eg, “telecare” [47], “telemedicine” [44], “telehealth” [41], “telehealth and remote monitoring” [29]), and most importantly in the types of interventions and technologies that qualify as HT. For instance, Chaudhry et al [26] argue in their review that there is no clear rational for excluding telephone-based interventions that use one-on-one telephone calls between nurses and patients, while other reviewers contend the opposite (eg, [31,32,48,49]. The protocol of our critical appraisal and in particular the examination of citation patterns revealed several discordant views between the included reviews on the inclusion, classification, and analysis of certain interventions. The majority of reviews strongly converged on the inclusion of interventions that were based on telemetry devices offering automated or message-based monitoring and transmission of physiologic signs or symptoms through communication networks (see Multimedia Appendix 3). However, there were important disagreements between reviews in the inclusion and analysis of other interventions such as stand-alone telephone support (52, 63), automated telephone calls, toll-free computerized voice answering systems (13, 31), videophone (70), television-based support (4), video-conferencing (46), and website-based support (35).

The following example provides a good illustration of the problem that currently exists and the consequences it has on the results and direct comparison of the results of HT reviews. An RCT that was included in 3 reviews of HT for heart failure [28,29,31], 2 MAs and 1 SR, comprised a control group of usual care and 2 intervention groups. The first intervention group was assigned to structured telephone support, while the second was assigned to videophone that did not involve any automated monitoring or transmission of vital signs and symptoms. The 2 reviews [28,29] considered the videophone intervention as home telemonitoring, while the third one did not [31]. The way the third review chose to treat this study was to combine both intervention groups into one and analyze them quantitatively as structured telephone support. This indicates that there is no commonly agreed upon definition of HT and its core properties. Future research should address this important issue by proposing and validating a taxonomy that would capture the different types/forms of HT and enable robust comparisons across trials.

### Quality Assessment of Included Studies

The validity of the results produced by prior reviews and the confidence in their conclusions depend to a large extent on the quality of the included studies. There is ample evidence showing that the scientific quality of primary studies is not always adequate and methodological flaws, when not identified and accounted for, may inflate or deflate the results of an SR [84-86]. Current guidelines [52,58] suggest two different quality assessments that must be performed by reviewers in each review: the methodological quality (or risk of bias) of the original studies and the quality of evidence [55] to indicate the extent to which we can be confident that an estimate of effect or the final conclusions of a review are correct across each outcome of interest. There also exist various strategies [58] that may be applied to incorporate the results of these assessments in the analysis and conclusions of the review. Unfortunately, our findings within the particular area of HT are rather disappointing and raise important concerns. Out of the 24 reviews, only 9 (38%) assessed the methodological quality of the included studies and 5 of them (21%) rated the overall quality of the available evidence. Furthermore, only 4 reviews factored the results of the quality assessment in their final conclusions. Therefore, the possibility that biased studies have inflated or deflated the results of prior reviews of HT cannot be ruled out.

### Selection and Justification of the Data Synthesis Method

Decisions concerning the selection of the data synthesis method that is most appropriate for addressing the research question(s) of the review require thoughtful consideration, as well as clinical judgment and should be based on explicit clinical and methodological criteria that minimize subjectivity as much as possible [68]. Based on the results of our evaluation, the rationale and criteria used to guide and support the decision of the researchers to synthesize the available evidence narratively or quantitatively was not always evident. Out of the 15 qualitative SRs, 8 (53%) provided some explanation for not conducting an MA, but even in these cases the criteria used to decide that studies were not clinically or methodologically similar enough to be pooled were not revealed. On their part, MAs of HT did not provide a rationale or a statement specifying what criteria were used to support the decision to combine statistically studies that may vary in terms of patients’ stages of severity, home telemonitoring approaches, implementation settings, and other important aspects. This finding indicates that most reviewers may use narrative synthesis or meta-analysis as a “default action”, based on methodological preferences or prior experiences rather than explicit and clinically relevant criteria that minimize subjectivity. However, it would be informative for future reviews to address this issue by clearly specifying any methods or specific rules (eg, logic models based on the PICO framework) that were used to guide the selection of a particular synthesis approach [6,22].

### Qualitative Synthesis of Studies

Authors employing narrative or qualitative synthesis should describe explicitly the analysis plan underpinning each stage of the evidence synthesis process, in order to clarify and support the logic that was used to reach the final conclusions. Presenting an analysis plan is of paramount importance and should be an integral part of the Methods section in future SRs of HT, as it clarifies the synthesis process, improves the transparency and reliability of the review, and acts as a safeguard against bias that can arise from placing inappropriate emphasis on the results of one study over another [87-89]. Such an analysis plan must incorporate among others appropriate techniques for the transformation of raw data to a common statistical or numerical measure (eg, risk ratios, mean differences) across studies selected for inclusion [87]. This will allow reviewers to develop meaningful summaries of effect sizes that can facilitate robust and transparent comparisons across the range of studied effects. Unfortunately, the majority of narrative SRs failed to meet these criteria and in most cases review authors tended to rely excessively on reported *P* values, which have a notorious record for being misleading, particularly in situations with small primary studies that have large within-study variance (ie, poor precision) and are not sufficiently powered to reach significant results [74]. Given the inherent risks of misinterpreting nonsignificant results as evidence of no effect, future SRs in this area should preferably synthesize the available data by estimating effect-sizes from each primary study (as it was done in one of the SRs [26]) rather than reported *P* values.

### Measuring Inconsistency of HT Effects in Meta-Analyses

One of the main objectives of the statistical methods used in MAs of HT interventions is to evaluate the dispersion among the results of the included studies, that is, the between-study heterogeneity in effect sizes, in order to assess the consistency of study findings. In light of observed heterogeneity, it is important to investigate and explain, whenever possible, what is causing it in order to increase scientific understanding and clinical relevance. With respect to the first goal, all 9 MAs included in our sample adhered closely to recommended guidelines and assessed formally the variability (heterogeneity) of the HT studies’ results by calculating either Cochran’s Q, I^2^, or both heterogeneity statistics in most cases. This was particularly encouraging and reflects a good practice that is generally consistent with other MAs in the health care domain [17]. However, the limitations of these metrics [64-67,70,71] and the uncertainty around the I^2^ point estimates, which can be expressed with 95% confidence intervals, were not considered in any of the included MAs. As a result, firm claims or inferences about the extent of inconsistencies in the HT effects between trials in most cases were stronger than the evidence allowed. Perhaps this limitation can be attributed to the fact that the Review Manager (RevMan) software, which was used in more than half of the MAs, does not provide users with a functionality to calculate the confidence intervals of I^2^. This is an issue that has also been highlighted by other researchers and communicated in hope that future updates of this software will make confidence intervals an integral part of I^2^ heterogeneity calculations [73].

Future MAs in this area should continue to use both statistics to measure the statistical significance and proportion of heterogeneity in the observed effects. However, the limitations of these metrics must be taken into consideration. The Q statistic is subject to the same caveats as all tests of significance and should always be interpreted with due caution based on the number of HT studies included in the analysis [70]. The I^2^ is not precise and hence, confidence intervals for I^2^ estimates should always be reported and interpreted carefully, as they are valuable for reflecting the uncertainty associated with the estimated ratio of true heterogeneity to total variation in the observed effects [69]. When the number of primary studies included in an MA is limited (eg, k<15) and the within-study variance is large, the I^2^ estimate should be interpreted with caution and any strong statements about the consistency of the observed HT effects “should be avoided or tempered appropriately, regardless of the results” [70]. Furthermore, when the sizes of HT effects vary substantially, as was the case with certain outcomes in some MAs (eg, [31,38]), this variance in the results should become the primary focus in the discussion of an MA and the summary effect should be less important or even not important at all [74].

### The Choice Between Fixed and Random Effects Meta-Analysis

When combining data from various HT studies, a major dilemma is to decide whether to perform a fixed or random effects meta-analysis. This decision is particularly important as the choice of model might affect the estimate of the effect size and, ultimately, the interpretation of the results [79,90]. A fixed effect MA of HT interventions is based on the premise that all studies included in the review are functionally identical and are estimating a common (fixed) treatment effect [74,91]. That is, there are no genuine differences; all factors that potentially could influence the observed effect size such as the nature of the intervention (eg, sophistication of the technology, frequency of data transmission, home visits, and educational support) are functionally the same in all studies. Thus, any observed between-study variation (ie, statistical heterogeneity) in the results is attributed only to sampling error. On the other hand, random effects MA is based on the premise that the observed estimates of treatment effect are not identical in the included HT studies but follow some distribution. That is, they vary from study to study because of genuine differences (eg, in the nature of the intervention) as well as sampling variability (chance). Studies may differ in the mix of participants (eg, stages of severity), the quality, or implementation of the intervention, and so on. Hence, each study is estimating a different underlying effect. As such, a fixed effect MA provides an estimate of a “common” treatment effect, while the summary result produced by random effects MA provides an estimate of the “average” treatment effect [74,90]. It is also important to note that from a statistical point of view, when the between-study variance (statistical heterogeneity) is 0%, random effects analysis is reduced and coincides with a fixed effect analysis, showing similar effects anyhow. However, in the presence of any between-study heterogeneity, fixed effect meta-analyses provide overly precise summary results with narrower confidence intervals than random effects meta-analyses [90]. As we present next, this can lead to spuriously lower levels of statistical significance for the summary effects and may wrongly imply that a “common” treatment effect exists when in reality there are real differences in treatment effects across studies [79,90].

Our evaluation revealed that the random effects model, which facilitates a broader outlook as it summarizes the distribution of the intervention effects across studies, appears to be the most preferable statistical model among MAs of HT interventions. Indeed, from a clinical perspective, the “one size fits all” approach of the fixed effect model appears to be difficult to justify. The participants and contextual characteristics of HT interventions in most cases differ in many practical ways that may have an impact on the results [22]. It is implausible that effect modifiers in HT studies such as the technology, patients, program characteristics, and risks of bias are functionally identical or equivalent across all the included trials. Both HT and usual care have evolved dramatically over the past 15 years and these temporal changes may have affected the results of the included trials, resulting in greater heterogeneity. Nevertheless, 2 MAs on heart failure [28,31] applied the fixed effect model, despite the functional differences between the trials and the presence of moderate (eg, I^2^>30%) to substantial (eg, I^2^>75%) statistical heterogeneity in the observed effects. The use of a fixed rather than a random effects model influenced their results, as it produced tighter confidence intervals and spuriously low levels of statistical significance for the effects of HT. Specifically, in the Cochrane review the effect estimate for all cause-hospitalization using the fixed effect model showed a statistically significant (*P*=.02) reduction of 9% favoring HT (RR 0.91, 95% CI 0.84-0.99). Whereas the random effects model yields a nonsignificant (*P*=.22) effect size of the same magnitude with a wider confidence interval (RR 0.91, 95% CI 0.78-1.06), reflecting the uncertainty behind the positive effects of HT on average. Similarly, in the MA by Clarke et al [28], the effect estimate for mortality using the fixed effect model shows a significant (*P*=.02) reduction of deaths by 23% in favor of HT (RR 0.77, 95% CI 0.61-0.97). However, the random effects model yields a more conservative and nonsignificant (*P=*.30) effect-size of 17% on average with wider confidence interval (RR 0.83, 95% CI 0.58-1.19), reflecting again that the underlying effect of HT may not always be positive across all patients and contexts. Given the clinical and methodological differences of the HT trials included in these 2 MAs, the use of the fixed effect model appears to be counterintuitive and the a priori assumptions that led to its selection should have been revisited, especially after the detection of statistical heterogeneity [74]. Future MAs of HT interventions should comply with methodological guidelines and describe explicitly the rationale and the criteria that were used to choose between fixed and random effects meta-analysis. Also, when the random effects approach is used, then the pooled results should be interpreted appropriately as the “average” effect of the HT intervention [90,91], as was done in one of the MAs [34] in our sample.

### Limitations

If we apply the critical review approach to our own review, we realize that a number of challenges were faced in the process of appraising the methodological quality of the included SRs and MAs, which may have in turn affected our findings. First, our appraisal was performed on the basis of the information reported, explicitly or implicitly, in each review. Therefore, as in all methodological quality or risk of bias assessments, the accuracy of the judgments made by the evaluators relies heavily on the reporting adequacy of the reviews. It is possible that the authors conducted their review more rigorously. However, being aware of the length restrictions imposed by the journals and in light of competing demands for reporting the main findings of their review, they might have decided to omit some methodological information that was perceived as subtle or less important to report. It is also possible that the peer-review process itself resulted in abbreviating the text to meet space limitations. One recommendation for future reviews to alleviate this issue is to provide essential details about the protocol of the review in an electronic version, as is the practice in several peer-reviewed journals today, to aid in understanding the systematic review process considered. On the other hand, peer-reviewed journals that have an interest in publishing SRs and MAs in the area of HT should devote space for publishing online supplementary material and adopt appropriate mechanisms for flagging problems with and allowing corrections of previous work, once errors or other important deficiencies have been identified [24]. Also, the research community must be prepared to validate the results of reviews, in order to correct them if necessary and the results must be published in such a way that will facilitate this process [24]. We conducted a post hoc analysis and found that out of the 16 journals in which the included reviews were published, 10 (63%) allowed the publication of online appendices but only 3 reviews provided an appendix or a supplement file.

Second, it is important to note that the findings of our evaluation are confined to the reviews that met our inclusion criteria described in the Methods section. Although our bibliographic search identified several “narrative reviews” that focus on the effectiveness of HT interventions on patients with various chronic diseases, when these were not self-identified as systematic or did not feature essential properties of an SR or MA, they were excluded from our study. This strict selection process may have contributed to an overestimation of the methodological quality of HT reviews as reflected by the R-AMSTAR instrument and our analysis. Also excluded were several reviews that provided an all-inclusive and mixed overview of HT interventions along with various other “remote monitoring” interventions (eg, structured telephone support and stand-alone video consultation), but did not make a clear distinction between them in the analysis of the results. Therefore, our findings are not generalizable to reviews in which HT was one among many other multidisciplinary interventions of remote patient monitoring, although most would agree that the highlighted methodological deficiencies have significant relevance and are applicable to these reviews as well.

### Conclusion

This study is the first attempt to evaluate the overall quality of prior SRs and MAs of HT interventions. The comprehensiveness of the search strategy used to identify relevant reviews, the duplicated process in relation to study selection, data extraction, and quality appraisal, as well as the use of a validated instrument that offers the ability to conduct an in-depth quality assessment, are key indicators of the methodological soundness of the present study.

The number of published SRs and MAs in the area of HT has substantially increased in the last decade offering to a wide range of health care stakeholders an extensive base of “large-scale evidence” from the synthesis of multiple primary studies on the clinical, behavioral, structural, and economic effects of HT for patients with chronic conditions. Yet, despite the significant body of knowledge that has been developed, wide acceptance by payers and care providers and integration of HT as an effective patient management approach remains problematic. This is mainly because the existing knowledge base still exhibits several important methodological weaknesses and research gaps.

Of utmost importance, our critical assessment revealed that the overall quality and rigor of existing SRs and MAs of HT interventions is highly variable, with no signs of improvement over time. An important number of reviews contain several common methodological shortcomings that impair their internal validity and limit their usefulness for clinical, educational, research, and policy purposes. As a result, a range of questions regarding the effectiveness of HT for chronic disease management remain unanswered, including which is the ideal and most effective combination of case management and remote monitoring, which behavior change techniques and modalities are most effective, whether the effectiveness of interventions is influenced by participant demographics and settings, and whether HT is an effective and viable solution from an economic point of view. We thus recommend that future reviews in this area improve their overall rigor as well as their reporting aspects by adhering closely to available methodological guidelines. More precisely, they should at least include the following elements: (1) clearly stated research question(s) explicitly describing the patient population, intervention, comparison intervention, and outcomes; (2) comprehensive and clearly stated search strategies; (3) formal appraisal of the validity of the primary studies (ie, risk of bias assessment) with appropriate attempts to explore the impact of studies with high risk of bias on the estimated effects of HT; and (4) more rigorous methods of data synthesis with transparent descriptions and justifications of the techniques or statistics used.

To conclude, it is our hope that this study will contribute to increase the overall quality of SRs and MAs in the HT area, as well as in the broader telehealth domain, by helping authors minimize diverse risks of biases and avoid previous methodological deficiencies. Nonetheless, we believe that building more rigorous and stronger evidence in the HT area will require unprecedented efforts by researchers, clinicians, funders, journal editors, and peer reviewers. Such efforts include but are not limited to the involvement of individuals with both clinical and methodological expertise in the conduct of SRs and MAs; amendments to the general instructions published by the journals with specific guidelines or links to methodological and reporting recommendations; the involvement of individuals in the peer-review process with prior experience and knowledge in the methodologies of SRs and MAs; and adoption of mechanisms to allow updates or corrections of online published material to address important deficiencies or even errors identified after publication.

## Multimedia Appendix 1

List of excluded articles.

## Multimedia Appendix 2

R-AMSTAR assessment of methodological rigor for each review.

## Multimedia Appendix 3

Citation analysis of systematic reviews and meta-analyses of home telemonitoring interventions for patients with heart failure.

## Abbreviations

GRADE: grading of recommendations assessment, development, and evaluation
HT: home telemonitoring
MA: meta-analysis
PICO: population, intervention, comparison, outcomes
PRISMA: preferred reporting items for systematic reviews and meta-analyses
QUOROM: quality of reporting of meta-analyses
R-AMSTAR: revised assessment of multiple systematic reviews
RCT: randomized controlled trial
SR: systematic review
