# Correction: Systematic Reviews and Meta-Analyses of Home Telemonitoring Interventions for Patients With Chronic Diseases: A Critical Assessment of Their Methodological Quality

**DOI:** 10.2196/jmir.3081

**Published:** 2013-11-07

**Authors:** Spyros Kitsiou, Guy Paré, Mirou Jaana

**Affiliations:** ^1^ Information Technology in Health Care HEC Montreal Montreal, QC Canada; ^2^ Telfer School of Management University of Ottawa Ottawa, ON Canada; ^3^ School of Business Lebanese American University Beirut Lebanon

In "Systematic Reviews and Meta-Analyses of Home Telemonitoring Interventions for Patients With Chronic Diseases: A Critical Assessment of Their Methodological Quality" (J Med Internet Res 2013;15(7):e150), there was an error in Table 6. After the second row of the subheading Clarke 2011 under the column “Low interval (95% CI)”, a 0 above the value 52 was inadvertently deleted in the copyediting process of the manuscript. As a result, all the values in the subsequent rows of “Low interval (95% CI)” in Table 6 were shifted upward, for example, the 52 in the third row belongs to the fourth row of the subheading Clarke 2011 under the column “Low interval (95% CI)”. In the last row of Table 6, the low interval should have been 0 and the high interval should have been 71 instead of being blank cells. These errors have been corrected in the online version of the paper on the JMIR website on November 4, 2013, together with publishing this correction notice. A correction notice has been sent to PubMed and the correct full-text has been resubmitted to Pubmed Central and other full-text repositories.

