# Supplementary material for: Systematic Reviews and Meta-Analyses of Home Telemonitoring Interventions for Patients With Chronic Diseases: A Critical Assessment of Their Methodological Quality
Source: J Med Internet Res. 2013 Jul 23;15(7):e150. doi: 10.2196/jmir.2770 (PMC3785977; doi:10.2196/jmir.2770)
Supplement: Supplementary file 1 [file jmir_v15i7e150_app1.pdf]

Multimedia Appendix 1. List of excluded articles.

Systematic reviews of other forms of telehealth interventions

1. Balas EA, Krishna S, Kretschmer RA, Cheek TR, Lobach DF, Boren SA. Computerized knowledge management in diabetes care. Med Care. 2004;42(6):610-621. [PubMed: 15167329]

2. Baron J, McBain H, Newman S. The impact of mobile monitoring technologies on glycosylated hemoglobin in diabetes: a systematic review. Journal of diabetes science and technology. Sep 2012;6(5):1185-1196. [PubMed: 23063046]

3. Cassimatis M, Kavanagh DJ. Effects of type 2 diabetes behavioural telehealth interventions on glycaemic control and adherence: a systematic review. J Telemed Telecare. 2012;18(8):447-450. Doi: 10.1258/jtt.2012.GTH105. [PubMed: 23209266]

4. Currell R, Urquhart C, Wainwright P, Lewis R. Telemedicine versus face to face patient care: effects on professional practice and health care outcomes. Cochrane Database Syst Rev. 2000(2):CD002098. Doi: 10.1002/14651858.cd002098. [PubMed: 10796678]

5. Durrani H, Khoja S. A systematic review of the use of telehealth in Asian countries. Journal of Telemedicine and Telecare. 2009;15(4):175-181. Doi: 10.1258/jtt.2009.080605.

6. Gaikwad R, Warren J. The role of home-based information and communications technology interventions in chronic disease management: a systematic literature review. Health Informatics Journal. 2009;15(2):122-146.Doi: 10.1177/1460458209102973.

7. Garcia-Lizana F, Sarria-Santamera A. New technologies for chronic disease management and control: a systematic review. J Telemed Telecare. 2007;13(2):62-68. Doi: 10.1258/135763307780096140. [PubMed: 17359568]

8. Hailey D, Roine R, Ohinmaa A. Systematic review of evidence for the benefits of telemedicine. J Telemed Telecare. 2002;8 Suppl 1:1-30. [PubMed: 12020415]

9. Heinzelmann PJ, Williams CM, Lugn NE, Kvedar JC. Clinical outcomes associated with telemedicine/telehealth. Telemed J E Health. Jun 2005;11(3):329-347. Doi: 10.1089/tmj.2005.11.329. [PubMed: 16035930]

10. Hersh W, Helfand M, Wallace J, et al. A systematic review of the efficacy of telemedicine for making diagnostic and management decisions. J Telemed Telecare. 2002;8(4):197-209. Doi: 10.1258/135763302320272167. [PubMed: 12217102]

11. Hersh WR, Hickam DH, Severance SM, Dana TL, Krages KP, Helfand M. Telemedicine for the medicare population: update. Evidence report/technology assessment. Feb 2006(131):1-41. [PubMed: 17900201]

12. Klersy C, De Silvestri A, Gabutti G, Regoli F, Auricchio A. A Meta-Analysis of Remote Monitoring of Heart Failure Patients. Journal of the American College of Cardiology. 10/27/ 2009;54(18):1683-1694. Doi: http://dx.doi.org/10.1016/j.jacc.2009.08.017.
13. McAlister FA, Stewart S, Ferrua S, McMurray JJ. Multidisciplinary strategies for the management of heart failure patients at high risk for admission: a systematic review of randomized trials. J Am Coll Cardiol. 2004;44(4):810-819. Doi: 10.1016/j.jacc.2004.05.055. [PubMed: 15312864]

14. McLean S, Chandler D, Nurmatov U, et al. Telehealthcare for asthma. Cochrane Database Syst Rev. 2010(10):CD007717. Doi: 10.1002/14651858.CD007717.pub2. [PubMed: 20927763]

15. McLean S, Nurmatov U, Liu JL, Pagliari C, Car J, Sheikh A. Telehealthcare for chronic obstructive pulmonary disease. Cochrane Database Syst Rev. 2011(7):CD007718. Doi: 10.1002/14651858.CD007718.pub2. [PubMed: 21735417]

16. Neubeck L, Redfern J, Fernandez R, Briffa T, Bauman A, Freedman SB. Telehealth interventions for the secondary prevention of coronary heart disease: a systematic review. European journal of cardiovascular prevention and rehabilitation : official journal of the European Society of Cardiology, Working Groups on Epidemiology & Prevention and Cardiac Rehabilitation and Exercise Physiology. Jun 2009;16(3):281-289. Doi: 10.1097/HJR.0b013e32832a4e7a. [PubMed: 19407659]

17. Polisena J, Coyle D, Coyle K, McGill S. Home telehealth for chronic disease management: a systematic review and an analysis of economic evaluations. Int J Technol Assess Health Care. 2009;25(3):339-349. Doi: 10.1017/s0266462309990201. [PubMed: 19619353]

18. Polisena J, Tran K, Cimon K, Hutton B, McGill S, Palmer K. Home telehealth for diabetes management: a systematic review and meta-analysis. Diabetes Obes Metab. 2009;11(10):913-930. Doi: 10.1111/j.1463-1326.2009.01057.x. [PubMed: 19531058]

19. Polisena J, Tran K, Cimon K, et al. Home telehealth for chronic obstructive pulmonary disease: a systematic review and meta-analysis. J Telemed Telecare. 2010;16(3):120-127. Doi: 10.1258/jtt.2009.090812. [PubMed: 20197355]

20. Radhakrishnan K, Jacelon C. Impact of telehealth on patient self-management of heart failure: a review of literature. J Cardiovasc Nurs. Jan-Feb 2012;27(1):33-43. Doi: 10.1097/JCN.0b013e318216a6e9. [PubMed: 21558862]

21. Roine R, Ohinmaa A, Hailey D. Assessing telemedicine: a systematic review of the literature. CMAJ : Canadian Medical Association journal = journal de l'Association medicale canadienne. Sep 18 2001;165(6):765-771. [PubMed: 11584564]

22. Tran K, Polisena J, Coyle D, et al. *Home telehealth for chronic disease management.* Ottawa, ON, Canada: Canadian Agency for Drugs and Technologies in Health, HTA issue 113;2008.

23. Verhoeven F, van Gemert-Pijnen L, Dijkstra K, Nijland N, Seydel E, Steehouder M. The contribution of teleconsultation and videoconferencing to diabetes care: a systematic

literature review. J Med Internet Res. 2007;9(5):e37. Doi: 10.2196/jmir.9.5.e37. [PubMed: 18093904]

24. Wainwright C, Wootton R. A Review of Telemedicine and Asthma. Disease Management & Health Outcomes. 2003;11(9):557-563. Doi: 10.2165/00115677-200311090-00003.

Non-systematic reviews

1. Fitzner K, Moss G. Telehealth-An Effective Delivery Method for Diabetes Self-Management Education? Population health management. Dec 5 2012. Doi: 10.1089/pop.2012.0054. [PubMed: 23216062]

2. Hasan A, Paul V. Telemonitoring in chronic heart failure. Eur Heart J. 2011;32(12):1457-1464. Doi: 10.1093/eurheartj/ehr005. [PubMed: 21289040]

3. Inglis SC, Clark RA, Cleland JG. Telemonitoring in patients with heart failure. The New England journal of medicine. Mar 17 2011;364(11):1078-1079; author reply 1079-1080. Doi: 10.1056/NEJMc1100395#SA2. [PubMed: 21410377]

4. Karunanithi M. Monitoring technology for the elderly patient. Expert review of medical devices. Mar 2007;4(2):267-277. Doi: 10.1586/17434440.4.2.267. [PubMed: 17359231]

5. Kleinpell R, Avitall B. Telemanagement in Chronic Heart Failure. Disease Management & Health Outcomes. 2005;13(1):43-52.

6. Konschak C, Flareau B. New frontiers in home telemonitoring. It's already here. Where are you? Journal of healthcare information management : JHIM. Summer 2008;22(3):16-23. Doi: [PubMed: 19267027]

7. Ni Scanaill C, Carew S, Barralon P, Noury N, Lyons D, Lyons GM. A review of approaches to mobility telemonitoring of the elderly in their living environment. Annals of biomedical engineering. Apr 2006;34(4):547-563. Doi: 10.1007/s10439-005-9068-2. [PubMed: 16550450]

8. Parati G, Omboni S. Role of home blood pressure telemonitoring in hypertension management: an update. Blood Press Monit. 2010;15(6):285-295. Doi: 10.1097/MBP.0b013e328340c5e4. [PubMed: 21084882]

9. Schmidt S, Schuchert A, Krieg T, Oeff M. Home telemonitoring in patients with chronic heart failure: a chance to improve patient care? Deutsches Arzteblatt international. Feb 2010;107(8):131-138. Doi: 10.3238/arztebl.2010.0131. [PubMed: 20300221]

10. Stoyanov N, Paul V. Clinical Use of Telemonitoring in Chronic Heart Failure: Keeping up with the Times or Misuse of Time? Current Heart Failure Reports. 2012;9(1):75-80.

Systematic reviews with multi-pathology patients

1. Barlow J, Singh D, Bayer S, Curry R. A systematic review of the benefits of home telecare for frail elderly people and those with long-term conditions. J Telemed Telecare. 2007;13(4):172-179. Doi: 10.1258/135763307780908058. [PubMed: 17565772]

2. Bensink M, Hailey D, Wootton R. A systematic review of successes and failures in home telehealth: preliminary results. Journal of Telemedicine and Telecare. 2006;12(suppl 3):8-16.Doi: 10.1258/135763306779380174.

3. Bowles KH, Baugh AC. Applying research evidence to optimize telehomecare. J Cardiovasc Nurs. 2007;22(1):5-15. [PubMed: 17224692]

4. Dellifraine JL, Dansky KH. Home-based telehealth: a review and meta-analysis. J Telemed Telecare. 2008;14(2):62-66. Doi: 10.1258/jtt.2007.070709. [PubMed: 18348749]

5. Jennett PA, Affleck Hall L, Hailey D, et al. The socio-economic impact of telehealth: a systematic review. J Telemed Telecare. 2003;9(6):311-320. Doi: 10.1258/135763303771005207. [PubMed: 14680514]

6. Jones JF, Brennan PF. Telehealth interventions to improve clinical nursing of elders. Annual review of nursing research. 2002;20:293-322. [PubMed: 12092513]

7. Rojas SV, Gagnon MP. A systematic review of the key indicators for assessing telehomecare cost-effectiveness. Telemed J E Health. Nov 2008;14(9):896-904. Doi: 10.1089/tmj.2008.0009. [PubMed: 19035798]

8. van den Berg N, Schumann M, Kraft K, Hoffmann W. Telemedicine and telecare for older patients--a systematic review. Maturitas. Oct 2012;73(2):94-114. Doi: 10.1016/j.maturitas.2012.06.010. [PubMed: 22809497]

Systematic reviews on other home telemonitoring topics

1. Bartoli L, Zanaboni P, Masella C, Ursini N. Systematic review of telemedicine services for patients affected by chronic obstructive pulmonary disease (COPD). Telemed J E Health. Nov 2009;15(9):877-883. Doi: 10.1089/tmj.2009.0044. [PubMed: 19919194]

2. Ciere Y, Cartwright M, Newman SP. A systematic review of the mediating role of knowledge, self-efficacy and self-care behaviour in telehealth patients with heart failure. J Telemed Telecare. 2012;18(7):384-391. Doi: 10.1258/jtt.2012.111009. [PubMed: 23019605]

Non English systematic reviews

1. Augustin U, Henschke C. [Does telemonitoring lead to health and economic benefits in patients with chronic heart failure? - a systematic review]. Gesundheitswesen (Bundesverband der Arzte des Offentlichen Gesundheitsdienstes (Germany)). Dec 2012;74(12):e114-121. Doi: 10.1055/s-0032-1309021. [PubMed: 22615027]

2. Paré G, Moqadem K, Pineau G, St-Hilaire C. *Revue systematique des effets de la telesurveillance a domicile dans le contexte du diabete, des maladies pulmonaires et des maladies cardiovasculaires. [Systematic review of the effects of home telemonitoring in the context of diabetes, pulmonary diseases and cardiovascular diseases]*. Montreal, Canada: Agence d'evaluation des technologies et des modes d'intervention en sante (AETMIS), ETMIS 5(3);2009.
