# Supplementary material for: Systematic Reviews and Meta-Analyses of Home Telemonitoring Interventions for Patients With Chronic Diseases: A Critical Assessment of Their Methodological Quality
Source: J Med Internet Res. 2013 Jul 23;15(7):e150. doi: 10.2196/jmir.2770 (PMC3785977; doi:10.2196/jmir.2770)
Supplement: Supplementary file 2 [file jmir_v15i7e150_app2.pdf]

**Multimedia Appendix 2.** R-AMSTAR assessment of methodological rigor for each SR and MA.

| Author (year) | Type of Synthesis | "A priori" design | Duplicated study selection and data extraction | Search comprehensiveness | Inclusion of grey literature | Included and excluded studies provided | Characteristics of the included studies | Quality assessment of the primary studies | Scientific quality of included studies used appropriately in formulating conclusions | Appropriateness of methods used to combine studies' findings | Publication bias | Conflict of Interest |
|---|---|---|---|---|---|---|---|---|---|---|---|---|
| **Heart Failure** | | **Q1** | **Q2** | **Q3** | **Q4** | **Q5** | **Q6** | **Q7** | **Q8** | **Q9** | **Q10** | **Q11** |
| Chaudhry 2007 [26] | SR | ab | No | abcd | b | ac | abc | ab | a | N/A | N/A | a |
| Clark 2007 [27] | MA | ab | abc | abcd | abd | ac | abc | ab | No | bcd | a | a |
| Clarke 2011 [28] | MA | ab | bc | abcd | No | No | No | No | No | bc | a | a |
| Dang 2009 [29] | SR | abc | No | abcd | b | abcd | abc | No | No | N/A | N/A | ab |
| Giamouzis 2012 [30] | SR | ab | No | abc | b | ac | abc | No | No | N/A | N/A | ab |
| Inglis 2010 [31] | MA | abc | abc | abcd | abc | abcd | abc | abcd | ab | bc | ab | a |
| Louis 2003 [32] | SR | abc | No | abcde | No | a | abc | No | No | N/A | N/A | No |
| Maric 2009 [33] | SR | ab | No | bcd | b | ac | abc | No | No | N/A | N/A | ab |
| Polisena 2010 [34] | MA | abc | abc | abc | bd | ac | abc | abcd | b | bcd | No | ac |
| Seto 2008 [35] | SR | abc | b | abc | b | a | abc | No | No | N/A | N/A | No |
| **Hypertension** | | **Q1** | **Q2** | **Q3** | **Q4** | **Q5** | **Q6** | **Q7** | **Q8** | **Q9** | **Q10** | **Q11** |
| AbuDagga 2010 [36] | SR | abc | a | abcd | b | a | abc | No | No | N/A | N/A | ab |
| Jaana 2007 [37] | SR | ab | No | abcd | b | abcd | abc | No | No | N/A | N/A | ab |
| Omboni 2011 [38] | MA | abc | abc | abcd | b | ac | ac | No | No | bcd | ab | a |
| Verberk 2011 [39] | MA | abc | No | acd | b | a | abc | No | No | bcd | ab | b |
| **Respiratory Conditions** | | **Q1** | **Q2** | **Q3** | **Q4** | **Q5** | **Q6** | **Q7** | **Q8** | **Q9** | **Q10** | **Q11** |
| Bolton 2011 [40] | SR | abc | abc | abde | bd | ac | abc | acd | ab | N/A | N/A | ab |
| Cox 2012 [41] | SR | ab | abc | abcde | No | ac | abc | ab | No | N/A | N/A | a |
| Franek 2012 [42] | SR | abc | No | abcd | b | abcd | abc | abcd | ab | N/A | N/A | ab |
| Jaana 2009 [43] | SR | abc | No | abcd | b | a | abc | No | No | N/A | N/A | ab |
| **Diabetes** | | **Q1** | **Q2** | **Q3** | **Q4** | **Q5** | **Q6** | **Q7** | **Q8** | **Q9** | **Q10** | **Q11** |
| Farmer 2005 [44] | MA | abc | ab | abcd | bd | ac | abc | ab | ab | bc | No | a |
| Jaana 2007 [45] | SR | abc | No | acd | b | a | abc | No | No | N/A | N/A | No |
| MAS 2009 [46] | MA | abc | No | abcd | b | abcd | abc | abcd | ab | bcd | No | ab |
| Montori 2004 [47] | MA | abc | abc | ab | No | a | abc | No | No | bcd | No | ab |
| **SRs covering all the above chronic diseases** | | **Q1** | **Q2** | **Q3** | **Q4** | **Q5** | **Q6** | **Q7** | **Q8** | **Q9** | **Q10** | **Q11** |
| Paré 2010 [48] | SR | abc | abc | abc | b | abcd | No | No | No | N/A | N/A | ab |
| Paré 2007 [49] | SR | ab | No | abcd | b | No | No | No | No | N/A | N/A | No |

SR: Systematic review; MA: Meta-analysis; N/A: non-applicable
