# Supplementary material for: Systematic Reviews and Meta-Analyses of Home Telemonitoring Interventions for Patients With Chronic Diseases: A Critical Assessment of Their Methodological Quality
Source: J Med Internet Res. 2013 Jul 23;15(7):e150. doi: 10.2196/jmir.2770 (PMC3785977; doi:10.2196/jmir.2770)
Supplement: Supplementary file 3 [file jmir_v15i7e150_app3.pdf]

Multimedia Appendix 3. Citation analysis of systematic reviews and meta-analyses of home telemonitoring interventions for patients with heart failure.

A bibliographic analysis was carried out by means of simple matrices to assess whether the identification of relevant primary studies in each systematic review and meta-analysis was comprehensive and the extent to which reviews overlapped.

Citation matrices that cross-linked individual reviews with the corresponding primary study identified and included in that review were generated separately for each chronic disease considered. Here, we present as an example, the citation patterns of reviews that focused on patients with heart failure.

Figure 1: Citation matrix of previously published RCTs of Home Telemonitoring interventions involving patients with Heart Failure (full peer reviewed publications, abstracts and non-English articles; duplicate publications have been merged)

Author - Publication date	Number of RCTs	Reported search range	Johnston 2000	Massie 2001	Bondmass 2001	Jerant 2001, 2003	De Lusignan 1999, 2001	Goldberg 2002, 2003	Woodend 2002, 2003, 2008	Benatar 2003	Barnason 2003	Artinian 2003	LaFramboise 2003	Noel 2004	Capomolla 2004	Cleland 2005; Robinson 2004	Weintraub 2005,2010	Finkelstein 2006	Kashem 2006	Villani 2007	Kielblock 2007	Blum 2007	Dansky 2008	Antonicelli 2008	Wakefield 2008	Balk 2008	Soran 2008	Schwarz 2008	Zugck 2008	Giordano 2009	Mortara 2009	Dar 2009	Scherr 2009	Tompkins 2010	Wade 2011	Koehler 2011	Dendale 2012
**Louis** - Oct 2003	7	1966 - 2002	■	A	A	■	■	A	A	░	░	░	░	X																							
**Chaudhry** - Feb 2007	4	1966 - Aug 2006				■		■		■						■		░	░	X																	
**Clark** - May 2007	5	Jan 2002 - May 2006*					■	■	■						■	■		░	░	X																	
**Paré** - May 2007	7	1990 - 2006					■	■		■					■	■				░	░	X															
**Seto** - Sep 2008	5	all dates - Nov 2007	■			■				■				■				■					░	X													
**Dang** - Oct 2009	9	1966 - Apr 2009	ex			■	ex	■	■	■				ex		■		ex	■	ex			■		■	ex				■			░	X			
**Polisena** - Mar 2010	11	1998 - 2008				■	■	■	■	■		■	■		■	■												■		■	░	░	░	X			
**Paré** - Apr 2010	12	1966 - 2008	ex				■	■	■	■	■		ex	ex		■		ex					■	■			■	■		░	░	░	░	X			
**Inglis** - Aug 2010	14	Jan 2002- Nov 2008**	ex			ex	■	■	■	ex		ex	ex	ex	■	■	ex	ex	ex	nE/A	nE	A	ex	■	TS	■	■	ex	A	■	■	░	░	░	░	X	
**Clarke** - Jan 2011	13	Jan 1969 - Oct 2009						■	■	■						■	■						■	■	■	■	■			■	■	■	░	░	░	X	
**Giamouzis** - June 2012	12	2001- Nov 2011						■								■								■			■			■	■	■	■	■	■	■	■

■ Primary study cited and included in the systematic review

nE Non English publication of primary study cited and included in the systematic review

A Study published as Absract

ex Study was cited as excluded because it did not meet the eligibility criteria

TS Study was interpreted and meta-analyzed as structured telephone support not home telemonitoring

░ Study published after the reported end date of the search for relevant articles

X Study published after publication date of the systematic review

* Update of the systematic review by Louis et al (2003)

** Update of the systematic review by Clark et al (2007)

Figure 2: Citation matrix of previously published RCTs of Home Telemonitoring interventions involving patients with Heart Failure (full peer reviewed English publications only; duplicate publications have been merged; abstracts are excluded)

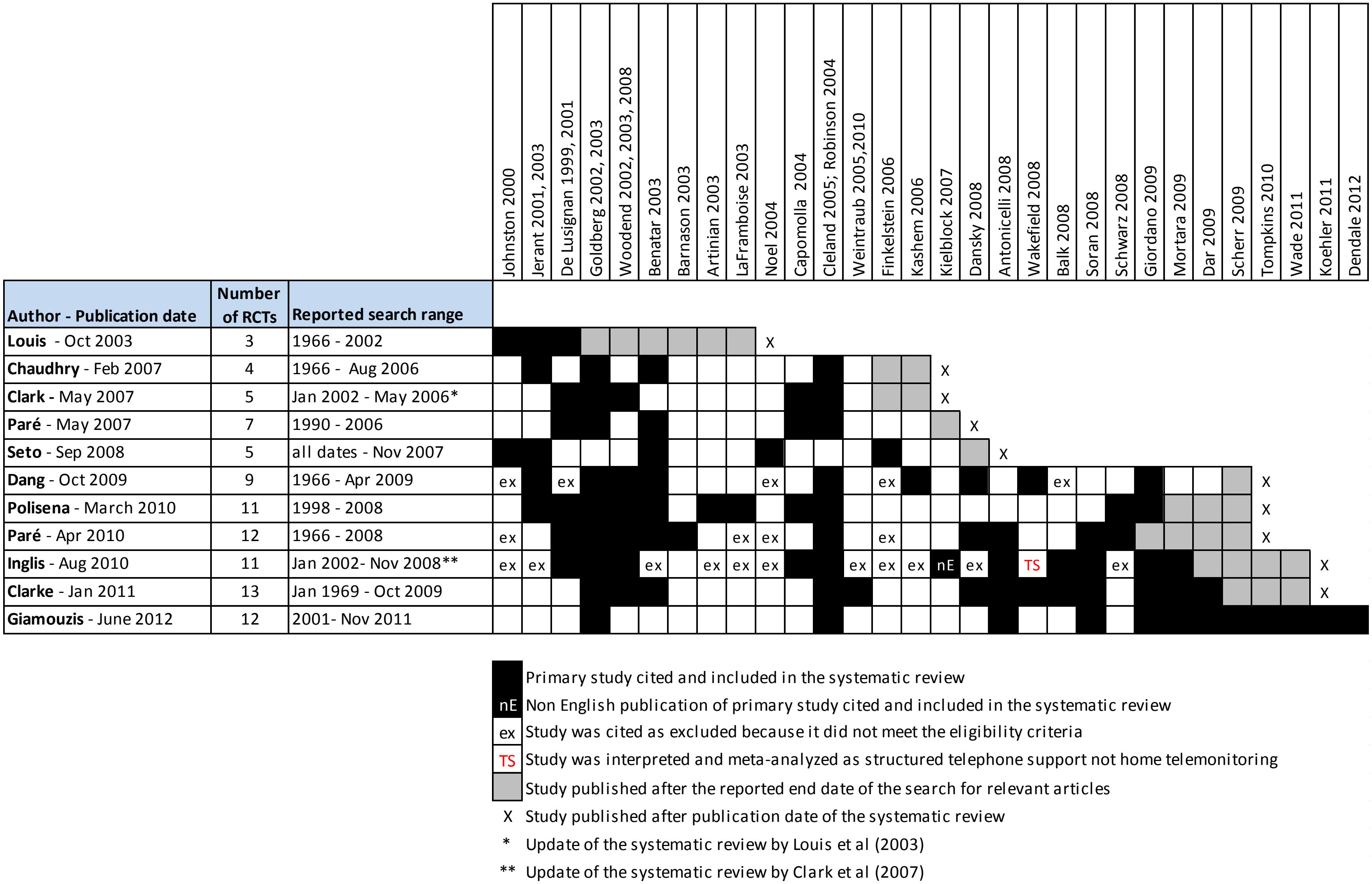

Figure 3: Citation matrix of previously published non-randomized and observational studies of Home Telemonitoring interventions involving patients with Heart Failure (full peer reviewed publications and abstracts)

Author - Publication date	Number of studies	Reported search range	Vincent 1997	Roglieri 1997	Shah 1998	Williams 1998	Bondmass 1999a	Chrysogelos 1999	Cordisco 1999	Heidenreich 1999	Bondmass 1999b	DeLusignan 2000	Lapworth 2000	Mehra 2000	Nanevicz 2000	Vaccaro 2001	Deering 2002	Ertle 2002	Feldman 2002	Knox 2002	Kesinger 2 002	Scalvini 2002	Macropoulos 2002	Wang 2002	Dimmick 2003	McManus 2004	Roth 2004	Bradford 2004, 2005	Schofield 2005	Kjellstrom 2005	Scalvini 2005	Lehmann 2006	Scherr 2006	Quinn 2006	Myers 2006	Seibert 2008
**Louis** - Oct 2003	17	1966 - 2002				A	A	A					A				A	A	A	A	A	A	A	A	X											
**Paré** - May 2007	9	1990 - 2006																																		X
**Seto** - Sep 2008	5	all dates - Nov 2007																																		
**Polisena** - March 2010	10	1998 - 2008																																		
**Paré** - Apr 2010	5	1966 - 2008	ex	ex																																

Primary study cited and included in the systematic review

A Study published as Absract

Study published after the end date of the search for relevant articles

ex Study was cited as excluded because it did not meet the eligibility criteria

X Study published after publication date of study in question

Figure 4: Citation matrix of previously published non-randomized and observational studies of Home Telemonitoring interventions involving patients with Heart Failure (full peer reviewed publications only)

Author - Publication date	Number of studies	Reported search range	Vincent 1997	Roglieri 1997	Shah 1998	Cordisco 1999	Heidenreich 1999	Bondmass 1999b	DeLusignan 2000	Mehra 2000	Nanevicz 2000	Vaccaro 2001	Dimmick 2003	McManus 2004	Roth 2004	Bradford 2004, 2005	Schofield 2005	Kjellstrom 2005	Scalvini 2005	Lehmann 2006	Scherr 2006	Quinn 2006	Myers 2006	Seibert 2008
**Louis** - Oct 2003	5	1966 - 2002		■	■	■	■		■				X											
**Paré** - May 2007	9	1990 - 2006	■		■	■	■				■				■		■	■	■					X
**Seto** - Sep 2008	5	all dates - Nov 2007											■			■			■	■			■	░
**Polisena** - March 2010	10	1998 - 2008				■		■		■		■		■	■		■					■	■	■
**Paré** - Apr 2010	5	1966 - 2008	ex	ex				■							■		■		■		■			

■ Primary study cited and included in the systematic review

A Study published as Absract

░ Study published after the end date of the search for relevant articles

ex Study was cited as excluded because it did not meet the eligibility criteria

X Study published after publication date of study in question

References of primary studies on heart failure included in the systematic reviews

1. Antonicelli R, Mazzanti I, Abbatecola AM, Parati G. Impact of home patient telemonitoring on use of β-blockers in congestive heart failure. Drugs & aging. 2010;27(10):801-805.Doi: 10.2165/11538210-000000000-00000. [PubMed: 20883060]

2. Antonicelli R, Testarmata P, Spazzafumo L, et al. Impact of telemonitoring at home on the management of elderly patients with congestive heart failure. Journal of telemedicine and telecare. 2008;14(6):300-305.Doi: 10.1258/jtt.2008.071213. [PubMed: 18776075]

3. Artinian NT, Harden JK, Kronenberg MW, et al. Pilot study of a Web-based compliance monitoring device for patients with congestive heart failure. Heart & lung : the journal of critical care. 2003;32(4):226-233. [PubMed: 12891162]

4. Balk AH, Davidse W, Dommelen Pv, et al. Tele-guidance of chronic heart failure patients enhances knowledge about the disease. A multi-centre, randomised controlled study. European journal of heart failure. 2008;10(1):1136-1142. Doi: 10.1016/j.ejheart.2008.08.003. [PubMed: 18790668]

5. Barnason S, Zimmerman L, Nieveen J, Schmaderer M, Carranza B, Reilly S. Impact of a home communication intervention for coronary artery bypass graft patients with ischemic heart failure on self-efficacy, coronary disease risk factor modification, and functioning. Heart & lung : the journal of critical care. 2003;32(3):147-158. [PubMed: 12827099]

6. Benatar D, Bondmass M, Ghitelman J, Avitall B. Outcomes of chronic heart failure. Archives of internal medicine. 2003;163(3):347-352.Doi: [PubMed: 12578516]

7. Blum K, Gottlieb SS. Morbidity and Mortality Benefits of Reliable Instrumental Support. Journal of Cardiac Failure. 2007;13(6):S164.Doi: 10.1016/j.cardfail.2007.06.581.

8. Bondmass M, Benetar J, Castro G, Avitall B. A prospective randomised study comparing outcomes and outpatient care delivery methods for chronic heart failure. Journal of the American College of Cardiology. 2001;37:1A-648A.

9. Bondmass M, Malhotra V, Castro G, Avitall B. The long term effect of a telemedicine intervention on heart failure admissions and length of stay. Journal of Cardiac Failure. 1999a;5(3):78. Doi: 10.1016/S1071-9164(99)91642-1.

10. Bondmass M, Bolger N, Castro G, Avitall B. The effect of physiologic home monitoring and telemanagement on chronic heart failure outcomes. The Internet Journal of Advanced Nursing Practice. 1999b;3(2).

11. Bradford WD, Kleit A, Krousel-Wood MA, Re RM. Comparing willingness to pay for telemedicine across a chronic heart failure and hypertension population. Telemedicine journal and e-health : the official journal of the American Telemedicine Association. 2005;11(4):430-438. Doi: 10.1089/tmj.2005.11.430. [PubMed: 16149888]

12. Bradford WD, Kleit AN, Krousel-Wood MA, Re RM. Willingness to pay for telemedicine assessed by the double-bounded dichotomous choice method. Journal of telemedicine and telecare. 2004;10(6):325-330.Doi: 10.1258/1357633042601991. [PubMed: 15603629]

13. Capomolla S, Pinna G, Larovere M, et al. Heart failure case disease management program: a pilot study of home telemonitoring versus usual care. European Heart Journal Supplements. 2004;6:F91-F98.Doi: 10.1016/j.ehjsup.2004.09.011.

14. Chrysogelos ET, Gemme D, Coleman K, Chung ES, Meyer TE. Telemonitoring devices further improve outcomes of a multidisciplinary heart failure outpatient program. Journal of Cardiac Failure. 1999;5(3):73.Doi: 10.1016/S1071-9164(99)91623-8.

15. Cleland JGF, Louis AA, Rigby AS, Janssens U, Balk AHMM. Noninvasive home telemonitoring for patients with heart failure at high risk of recurrent admission and death: the Trans-European Network-Home-Care Management System (TEN-HMS) study. Journal of the American College of Cardiology. 2005;45(10):1654-1664. Doi: 10.1016/j.jacc.2005.01.050. [PubMed: 15893183]

16. Cordisco ME, Benjaminovitz A, Hammond K, Mancini D. Use of telemonitoring to decrease the rate of hospitalization in patients with severe congestive heart failure. The American journal of cardiology. 1999;84(7):860-862, A868. Doi: [PubMed: 10513789]

17. Dansky KH, Vasey J, Bowles K. Impact of telehealth on clinical outcomes in patients with heart failure. Clinical nursing research. 2008;17(3):182-199. Doi: 10.1177/1054773808320837. [PubMed: 18617707]

18. Dar O, Riley J, Chapman C, et al. A randomized trial of home telemonitoring in a typical elderly heart failure population in North West London: results of the Home-HF study. European journal of heart failure. 2009;11(3):319-325. Doi: 10.1093/eurjhf/hfn050. [PubMed: 19174529]

19. de Lusignan S, Althans A, Wells S, Johnson P, Vandenburg M, Robinson J. A pilot study of radiotelemetry for continuous cardiopulmonary monitoring of patients at home. Journal of telemedicine and telecare. 2000;6 Suppl 1:S119-122. [PubMed: 10793994]

20. de Lusignan S, Meredith K, Wells S, Leatham E, Johnson P. A controlled pilot study in the use of telemedicine in the community on the management of heart failure--a report of the first three months. Studies in health technology and informatics. 1999;64:126-137. [PubMed: 10747531]

21. de Lusignan S, Wells S, Johnson P, Meredith K, Leatham E. Compliance and effectiveness of 1 year's home telemonitoring. The report of a pilot study of patients with chronic heart failure. European journal of heart failure. 2001;3(6):723-730. [PubMed: 11738225]

22. Deering M, Baines B, Christianson C, Milner J. Patients and providers evaluate daily home weight and symptom monitoring for CHF management. Journal of cardiac failure. 2002;8(4):S97.

23. Dendale P, De Keulenaer G, Troisfontaines P, et al. Effect of a telemonitoring-facilitated collaboration between general practitioner and heart failure clinic on mortality and rehospitalization rates in severe heart failure: the TEMA-HF 1 (TElemonitoring in the

MAnagement of Heart Failure) study. European journal of heart failure. 2012;14(3):333-340. Doi: 10.1093/eurjhf/hfr144. [PubMed: 22045925]

24. Dimmick SL, Burgiss SG, Robbins S, Black D, Jarnagin B, Anders M. Outcomes of an integrated telehealth network demonstration project. Telemedicine journal and e-health : the official journal of the American Telemedicine Association. 2003;9(1):13-23. Doi: 10.1089/153056203763317611. [PubMed: 12699604]

25. Ertle D, Litman G. Hospital outpatient disease management initiative achieves marked reduction in inpatient admissions and costs for congestive heart failure. Journal of cardiac failure. 2002;8(4):S90.

26. Feldman C, Milstein S, Cinnamond M, GL M. A protocol driven diuretic dosing system based on bioelectric impedance reduces acute episodes of fluid overload in patients with severe heart failure while minimizing physician management time. Journal of cardiac failure. 2002;8(4):S84.

27. Finkelstein SM, Speedie SM, Potthoff S. Home telehealth improves clinical outcomes at lower cost for home healthcare. Telemedicine journal and e-health : the official journal of the American Telemedicine Association. 2006;12(2):128-136. Doi: 10.1089/tmj.2006.12.128. [PubMed: 16620167]

28. Giordano A, Scalvini S, Zanelli E, et al. Multicenter randomised trial on home-based telemanagement to prevent hospital readmission of patients with chronic heart failure. International journal of cardiology. 2009;131(2):192-199. Doi: 10.1016/j.ijcard.2007.10.027. [PubMed: 18222552]

29. Goldberg L, Piette J, Walsh M, Frank T, Jaski B. A daily electronic home monitoring system in patients with advanced heart failure improves survival: The WHARF (Weight Monitoring in Heart Failure) trial. Journal of cardiac failure. 2002;8(4):S54.

30. Goldberg LR, Piette JD, Walsh MN, et al. Randomized trial of a daily electronic home monitoring system in patients with advanced heart failure: the Weight Monitoring in Heart Failure (WHARF) trial. American heart journal. 2003;146(4):705-712. Doi: 10.1016/S0002-8703(03)00393-4. [PubMed: 14564327]

31. Heidenreich PA, Ruggerio CM, Massie BM. Effect of a home monitoring system on hospitalization and resource use for patients with heart failure. American heart journal. 1999;138(4 Pt 1):633-640. [PubMed: 10502207]

32. Jerant AF, Azari R, Martinez C, Nesbitt TS. A randomized trial of telenursing to reduce hospitalization for heart failure: patient-centered outcomes and nursing indicators. Home health care services quarterly. 2003;22(1):1-20. Doi: 10.1300/J027v22n01_01. [PubMed: 12749524]

33. Jerant AF, Azari R, Nesbitt TS. Reducing the cost of frequent hospital admissions for congestive heart failure: a randomized trial of a home telecare intervention. Medical care. 2001;39(11):1234-1245. [PubMed: 11606877]

34. Johnston B, Wheeler L, Deuser J, Sousa KH. Outcomes of the Kaiser Permanente Tele-Home Health Research Project. Archives of family medicine. 2000;9(1):40-45.Doi: [PubMed: 10664641]

35. Kashem A, Droogan MT, Santamore WP, et al. Web-based Internet telemedicine management of patients with heart failure. Telemedicine journal and e-health : the official journal of the American Telemedicine Association. 2006;12(4):439-447.Doi: 10.1089/tmj.2006.12.439. [PubMed: 16942416]

36. Kesinger T, Gilani S, Jennison S. Electronic home monitoring reduces hospital admission, length of stay and readmission frequency in a selected heart failure population. Journal of cardiac failure. 2002;8(4):S94.

37. Kielblock B, Frye C, Kottmair S, Hudler T, Siegmund-Schultze E, Middeke M. [Impact of telemetric management on overall treatment costs and mortality rate among patients with chronic heart failure]. Deutsche medizinische Wochenschrift (1946). 2007;132(9):417-422.Doi: 10.1055/s-2007-970350. [PubMed: 17315117]

38. Kjellström B, Igel D, Abraham J, Bennett T, Bourge R. Trans-telephonic monitoring of continuous haemodynamic measurements in heart failure patients. Journal of telemedicine and telecare. 2005;11(5):240-244.Doi: 10.1258/1357633054471795. [PubMed: 16035966]

39. Knox DA, Mueller TM, Vuckovic KM, Acker K. Remote titration of beta-blocker therapy for heart failure by advanced practice nurses, titration protocols, and daily patient telemanagment. Journal of cardiac failure. 2002;8(4):S83.

40. Koehler F, Winkler S, Schieber M, et al. Impact of remote telemedical management on mortality and hospitalizations in ambulatory patients with chronic heart failure: the telemedical interventional monitoring in heart failure study. Circulation. 2011;123(17):1873-1880.Doi: 10.1161/CIRCULATIONAHA.111.018473. [PubMed: 21444883]

41. LaFramboise LM, Todero CM, Zimmerman L, Agrawal S. Comparison of Health Buddy with traditional approaches to heart failure management. Family & community health. 2003;26(4):275-288.Doi: [PubMed: 14528134]

42. Lapworth DJ, Dibiase A. Decreased hospitalizations using a home based electronic weight monitoring system as part of a comprehensive heart failure program. Journal of cardiac failure. 2000;6:69.

43. Lehmann CA, Mintz N, Giacini JM. Impact of Telehealth on Healthcare Utilization by Congestive Heart Failure Patients. Disease Management and Health Outcomes. 2006;14(3).

44. Macropoulos LR, Knoop JD. CHF hospital admissions reduced by 57% (0.234 PPPY) in medicare population and 48% (0.299 PPPY) in commercial population using advanced home monitoring program in large patient population. Journal of Cardiac Failure. 2002;9(5):S103.Doi: 10.1016/S1071-9164(03)00178-7.

45. Massie B, West J, Van Ostaeyen D, Salbalvaro A. A controlled trial of heart failure management programs. Journal of the American College of Cardiology. 2001;37:1A-648A.

46. McManus SG. A Telehealth Program to Reduce Readmission Rates Among Heart Failure Patients: One Agency's Experience. Home Health Care Management & Practice. 2004;16(4):250-254.Doi: 10.1177/1084822303262542.

47. Mehra MR, Uber PA, Chomsky DB, Oren R. Emergence of electronic home monitoring in chronic heart failure: rationale, feasibility, and early results with the HomMed Sentry-Observer system. Congestive heart failure (Greenwich, Conn.). 2000;6(3):137-139. [PubMed: 12029180]

48. Mortara A, Pinna GD, Johnson P, et al. Home telemonitoring in heart failure patients: the HHH study (Home or Hospital in Heart Failure). European journal of heart failure. 2009;11(3):312-318.Doi: 10.1093/eurjhf/hfp022. [PubMed: 19228800]

49. Myers S. Impact of Home-Based Monitoring on the Care of Patients with Congestive Heart Failure. Home Health Care Management & Practice. 2006;18(6):444-451.Doi: 10.1177/1084822306289991.

50. Nanevicz T, Piette J, Zipkin D, et al. The feasibility of a telecommunications service in support of outpatient congestive heart failure care in a diverse patient population. Congestive heart failure (Greenwich, Conn.). 2000;6(3):140-145. [PubMed: 12029181]

51. Noel HC, Vogel DC, Erdos JJ, Cornwall D, Levin F. Home telehealth reduces healthcare costs. Telemedicine journal and e-health : the official journal of the American Telemedicine Association. 2004;10(2):170-183. [PubMed: 15319047]

52. Quinn C. Low-technology heart failure care in home health: improving patient outcomes. Home healthcare nurse. 2006;24(8):533-540. [PubMed: 17012959]

53. Robinson S, Stroetmann K, Stroetmann V. Tele-homecare for chronically ill patients: Improved outcomes and new developments. The Journal on Information Technology in Healthcare. 2004;2(4):251-262.

54. Roglieri JL, Futterman R, McDonough KL, et al. Disease management interventions to improve outcomes in congestive heart failure. The American journal of managed care. 1997;3(12):1831-1839. [PubMed: 10178473]

55. Roth A, Kajiloti I, Elkayam I, Sander J, Kehati M, Golovner M. Telecardiology for patients with chronic heart failure: the 'SHL' experience in Israel. International journal of cardiology. 2004;97(1):49-55.Doi: 10.1016/j.ijcard.2003.07.030. [PubMed: 15336806]

56. Scalvini S, Capomolla S, Zanelli E, et al. Effect of home-based telecardiology on chronic heart failure: costs and outcomes. Journal of telemedicine and telecare. 2005;11 Suppl 1(1):16-18.Doi: 10.1258/1357633054461688. [PubMed: 16035980]

57. Scalvini S, Zanelli E, Volterrani M, Benigno M. Effect of a home based telecardiology on chronic heart failure outcomes: a case control pilot study. ESC Congress. 2002, 31 August – 4 September, Berlin, Germany.

58. Scherr D, Kastner P, Kollmann A, et al. Effect of home-based telemonitoring using mobile phone technology on the outcome of heart failure patients after an episode of acute decompensation: randomized controlled trial. Journal of medical Internet research. 2009;11(3):e34. Doi: 10.2196/jmir.1252. [PubMed: 19687005]

59. Scherr D, Zweiker R, Kollmann A, Kastner P, Schreier G, Fruhwald FM. Mobile phone-based surveillance of cardiac patients at home. Journal of telemedicine and telecare. 2006;12(5):255-261. Doi: 10.1258/135763306777889046. [PubMed: 16848939]

60. Schofield RS, Kline SE, Schmalfuss CM, et al. Early outcomes of a care coordination-enhanced telehome care program for elderly veterans with chronic heart failure. Telemedicine journal and e-health : the official journal of the American Telemedicine Association. 2005;11(1):20-27. Doi: 10.1089/tmj.2005.11.20. [PubMed: 15785217]

61. Schwarz KA, Mion LC, Hudock D, Litman G. Telemonitoring of heart failure patients and their caregivers: a pilot randomized controlled trial. Progress in cardiovascular nursing. 2008;23(1):18-26. [PubMed: 18326990]

62. Seibert PS, Whitmore TA, Patterson C, et al. Telemedicine facilitates CHF home health care for those with systolic dysfunction. International journal of telemedicine and applications. 2008:235031. Doi: 10.1155/2008/235031. [PubMed: 18369411]

63. Shah NB, Der E, Ruggerio C, Heidenreich PA, Massie BM. Prevention of hospitalizations for heart failure with an interactive home monitoring program. American heart journal. 1998;135(3):373-378. Doi: [PubMed: 9506321]

64. Soran OZ, Piña IL, Lamas GA, et al. A randomized clinical trial of the clinical effects of enhanced heart failure monitoring using a computer-based telephonic monitoring system in older minorities and women. Journal of cardiac failure. 2008;14(9):711-717.Doi: 10.1016/j.cardfail.2008.06.448. [PubMed: 18995174]

65. Tompkins C, Orwat J. A randomized trial of telemonitoring heart failure patients. Journal of healthcare management / American College of Healthcare Executives. 2010;55(5):312-322; discussion 322-313. [PubMed: 21077581]

66. Vaccaro J, Cherry J, Harper A, O'Connell M. Utilization Reduction, Cost Savings, and Return on Investment for the PacifiCare Chronic Heart Failure Program, "Taking Charge of Your Heart Health". Disease Management. 2001;4(3):131-142.Doi: 10.1089/10935070152596052.

67. Villani A, Malfatto G, Della Rosa F, et al. [Disease management for heart failure patients: role of wireless technologies for telemedicine. The ICAROS project]. Giornale italiano di cardiologia (2006). 2007;8(2):107-114. [PubMed: 17402355]

68. Vincent JA, Cavitt DL, Karpawich PP. Diagnostic and Cost Effectiveness of Telemonitoring the Pediatric Pacemaker Patient. Pediatric Cardiology. 1997;18(2):86-90.Doi: 10.1007/s002469900121.

69. Wade MJ, Desai AS, Spettell CM, et al. Telemonitoring with case management for seniors with heart failure. The American journal of managed care. 2011;17(3):e71-79.Doi: [PubMed: 21504262]

70. Wakefield BJ, Ward MM, Holman JE, et al. Evaluation of home telehealth following hospitalization for heart failure: a randomized trial. Telemedicine journal and e-health : the official journal of the American Telemedicine Association. 2008;14(8):753-761.Doi: 10.1089/tmj.2007.0131. [PubMed: 18954244]

71. Wang L, Yu C-M, Chau E, Lam W-F. Feasibility of predicting CHF hospitalization using pacemaker-based impedance sensor in CHF patients. Journal of cardiac failure. 2002;8(4):S81.

72. Weintraub AR, Kimmelstiel C, Levine D, et al. A Multicenter Randomized Controlled Comparison of Telephonic Disease Management vs. Automated Home Monitoring in Patients Recently Hospitalized with Heart Failure: Span-CHF II Trial. Journal of Cardiac Failure. 2005;11(9):720.Doi: 10.1016/j.cardfail.2005.10.009.

73. Weintraub A, Gregory D, Patel AR, et al. A multicenter randomized controlled evaluation of automated home monitoring and telephonic disease management in patients recently hospitalized for congestive heart failure: the SPAN-CHF II trial. Journal of cardiac failure. 2010;16(4):285–92. doi:10.1016/j.cardfail.2009.12.012. [PubMed: 20350694]

74. Williams RE, Keiler L, Sprang M. Telemanagement of congestive heart failure: results of daily weight and symptom tracking. American College of Cardiology Annual Scientific Session. 1998. Abstract: 977-177

75. Woodend A, Fraser M, Sherrard H, Stueve L. Readmission and quality of life: the impact of telehome care in heart failure. Journal of cardiac failure. 2002;8(4):S98.

76. Woodend A, Sherrard H, Fraser M, et al. Getting Connected: Telehome Care for Patients. Canadian Journal of Home Economics. 2003;52(2):19-28.

77. Woodend AK, Sherrard H, Fraser M, Stuewe L, Cheung T, Struthers C. Telehome monitoring in patients with cardiac disease who are at high risk of readmission. Heart & lung : the journal of critical care. 2008;37(1):36-45.Doi: 10.1016/j.hrtlng.2007.04.004. [PubMed: 18206525]

78. Zugck C, Frankeinstein L, Nelles M, et al. Telemedicine reduces hospitalisation rates in patients with chronic heart failure - results of the randomized HiTel trial. European Journal of Heart Failure Supplements. 2008;7:9-9.Doi: 10.1016/S1567-4215(08)60024-7.
